# Efficient hepatocyte differentiation of primary human hepatocyte-derived organoids using three dimensional nanofibers (HYDROX) and their possible application in hepatotoxicity research

**DOI:** 10.1038/s41598-024-61544-y

**Published:** 2024-05-13

**Authors:** Yanran Tong, Yukiko Ueyama-Toba, Jumpei Yokota, Hayato Matsui, Masaki Kanai, Hiroyuki Mizuguchi

**Affiliations:** 1 Laboratory of Biochemistry and Molecular Biology, Graduate School of Pharmaceutical Sciences, Osaka University, 1-6 Yamadaoka, Suita, Osaka 565-0871 Japan; 2 Laboratory of Functional Organoid for Drug Discovery, National Institute of Biomedical Innovation, Health and Nutrition, Osaka, 567-0085 Japan; 3 Integrated Frontier Research for Medical Science Division, Institute for Open and Transdisciplinary Research Initiatives, Osaka University, Osaka, 565-0871 Japan; 4 Bio-Industry Unit, Technology Research Laboratory, Shimadzu Corporation, Kyoto, 619-0237 Japan; 5 Cell Business Unit, Diagnostics Management Department, Analytical and Measuring Instruments Division, Shimadzu Corporation, Kyoto, 619-0237 Japan; 6 Global Center for Medical Engineering and Informatics, Osaka University, Osaka, 565-0871 Japan; 7 Center for Infectious Disease Education and Research (CiDER), Osaka University, Osaka, 565-0871 Japan

**Keywords:** Stem-cell differentiation, Toxicology

## Abstract

Human liver organoids are in vitro three dimensionally (3D) cultured cells that have a bipotent stem cell phenotype. Translational research of human liver organoids for drug discovery has been limited by the challenge of their low hepatic function compared to primary human hepatocytes (PHHs). Various attempts have been made to develop functional hepatocyte-like cells from human liver organoids. However, none have achieved the same level of hepatic functions as PHHs. We here attempted to culture human liver organoids established from cryopreserved PHHs (PHH-derived organoids), using HYDROX, a chemically defined 3D nanofiber. While the proliferative capacity of PHH-derived organoids was lost by HYDROX-culture, the gene expression levels of drug-metabolizing enzymes were significantly improved. Enzymatic activities of cytochrome P450 3A4 (CYP3A4), CYP2C19, and CYP1A2 in HYDROX-cultured PHH-derived organoids (Org-HYDROX) were comparable to those in PHHs. When treated with hepatotoxic drugs such as troglitazone, amiodarone and acetaminophen, Org-HYDROX showed similar cell viability to PHHs, suggesting that Org-HYDROX could be applied to drug-induced hepatotoxicity tests. Furthermore, Org-HYDROX maintained its functions for up to 35 days and could be applied to chronic drug-induced hepatotoxicity tests using fialuridine. Our findings demonstrated that HYDROX could possibly be a novel biomaterial for differentiating human liver organoids towards hepatocytes applicable to pharmaceutical research.

## Introduction

Organoids are three-dimensional (3D) structured cells derived from various cell types including somatic stem cells and differentiated cells^1,2^. Organoids recapitulate the in vitro characteristics of biological tissues or organs through self-organization, and they are shown to be relatively genetically stable^3,4^. Thus, unlike conventional two-dimensional (2D) cultured cells, organoids are anatomically similar to biological organs, allowing the function of human organs to be studied in greater detail in vitro than previously possible.

Huch et al. reported that human adult bile duct cells derived from human biopsies could be expanded in vitro as human liver organoids, later termed intrahepatic cholangiocyte organoids (ICOs)^2,5^. They also defined the culture conditions which allow long-term expansion of human liver organoids^4^. Thereafter, several groups clarified that human liver organoids have a bipotent stem cell phenotype, and therefore could differentiate into functional hepatocytes, which in turn suggested their potential as a novel hepatocyte model^6–8^. In addition, Huch et al. defined a differentiation medium that allow human liver organoids to differentiate into functional hepatocyte-like cells with higher hepatic functions than a human hepatoma cell line (HepG2)^4^. Broutier et al. demonstrated that when human liver organoids established from isolated human ducts or sorted ductal cells were cultured in hepatocyte differentiation medium, more than 60% of cells became positive for two major hepatocyte markers, albumin (ALB) and hepatocyte nuclear factor 4 alpha (HNF4a)^9^. Despite such scientific breakthroughs, translation of human liver organoids as a novel hepatocyte model in the drug discovery field has remained a challenge because their hepatic function is still too low and not comparable to that of primary human hepatocytes (PHHs)^7^, the gold-standard hepatocytes widely used in pharmaceutical research, regenerative medicine and tissue engineering.

In addition to optimizing the culture medium for human liver organoids to promote their hepatic differentiation, culture substrates for human liver organoids have been studied to further enable translational research. Generally, organoids are embedded and cultured in Matrigel, a gelatinous protein mixture composed of various elements including laminin, collagen type IV and growth factors extracted from Engelbreth-Holm-Swarm mouse sarcoma, which provides the cells with a bioactive environment to proliferate and self-organize^10,11^. However, this murine-derived Matrigel is poorly defined and has batch-to-batch variability of up to 50%^10,12^. Hence, various synthetic hydrogels have been developed as promising culture substrates for organoids, since they can be tuned and chemically modified with relative ease^13–15^. Willemse et al*.* succeeded in culturing and expanding human liver organoids in liver extracellular matrix-derived hydrogel. They also showed that human liver organoids maintained in the hydrogel could differentiate toward hepatocyte-like cells expressing *ALB*, when cultured in differentiation medium^16^. Ye et al. showed that human liver organoids cultured in a novel hydrogel based on polyisocyanopeptides differentiated toward a functional hepatocyte-like phenotype when cultured in differentiation medium^17^. Moreover, Krüger et al*.* investigated a cellulose nanofibril hydrogel that can differentiate human liver organoids towards hepatocyte-like cells with hepatic functions equivalent to or better than those of the human liver organoids cultured with Matrigel, when cultured in differentiation medium^18^. Collectively, these studies indicate that synthetic hydrogels themselves support organoid growth as an alternative to Matrigel, but often do not benefit the maturation of human liver organoids toward hepatocytes. In addition, even when these human liver organoids were cultured with differentiation medium, their hepatic function was not comparable to that in human liver or PHHs. Therefore, in order to achieve efficient clinical research using human liver organoids, a novel chemically modified human liver organoids culture system to obtain highly functional hepatocyte-like cells is desired.

Our group previously developed an easy-to-use biodegradable amphiphilic polymer consisting of poly(sarcosine) and poly(L-lactic acid) (PSar-PLLA)^19,20^. We developed a 3D nanofiber biomaterial based on this polymer, named HYDROX, and showed that it was applicable to the 3D culture of various cell types. In addition, we demonstrated that the HYDROX-culture system could be used to promote the differentiation and maturation of human induced pluripotent stem (iPS) cell-derived hepatocyte-like cells^21^. Given these advantages, we hypothesized that HYDROX could be a promising xeno-free biomaterial for human liver orgnaoids culture and differentiation toward hepatocytes.

In this study, we attempted to apply the HYDROX-culture system to human liver organoids that were established from commercially available cryopreserved PHHs (PHH-derived organoids). We then examined the histological characteristics of the resulting HYDROX-cultured PHH-derived organoids (Org-HYDROX). We also investigated various hepatic functions of Org-HYDROX by examining the gene expression levels and drug metabolic activities of major hepatocyte markers, followed by a comprehensive gene expression analysis. The applicability of Org-HYDROX to a drug-induced hepatotoxicity test was also investigated. Finally, we attempted a long-term culture of Org-HYDROX and tested its ability to predict chronic drug-induced hepatotoxicity.

## Results

### Cultivation of PHH-derived organoids using the HYDROX-culture system

Human liver organoids were established from PHHs (lot HC4-24) and cultured with the medium for cholangiocyte organoids according to the report of Huch et al.^5^ Phase contrast microscopic images revealed that the established organoids formed a spherical structure (Fig. S1A). We confirmed that the gene expression levels of cholangiocyte markers (*Epithelial cell adhesion molecule (EpCAM)*, *cytokeratin7 (CK7)*, *cytokeratin19 (CK19)*) were upregulated to the same level as those of HuCCT1 cells, a human bile duct carcinoma cell line (Fig. S1B), whereas hepatocyte markers (*cytochrome P450 family 3 subfamily A member 4 (CYP3A4)*, *albumin (ALB)*) decreased (Fig. S1C) compared to those of PHHs, as previously reported for the human liver organoids produced by Huch et al.^4,5^

**Figure 1:**
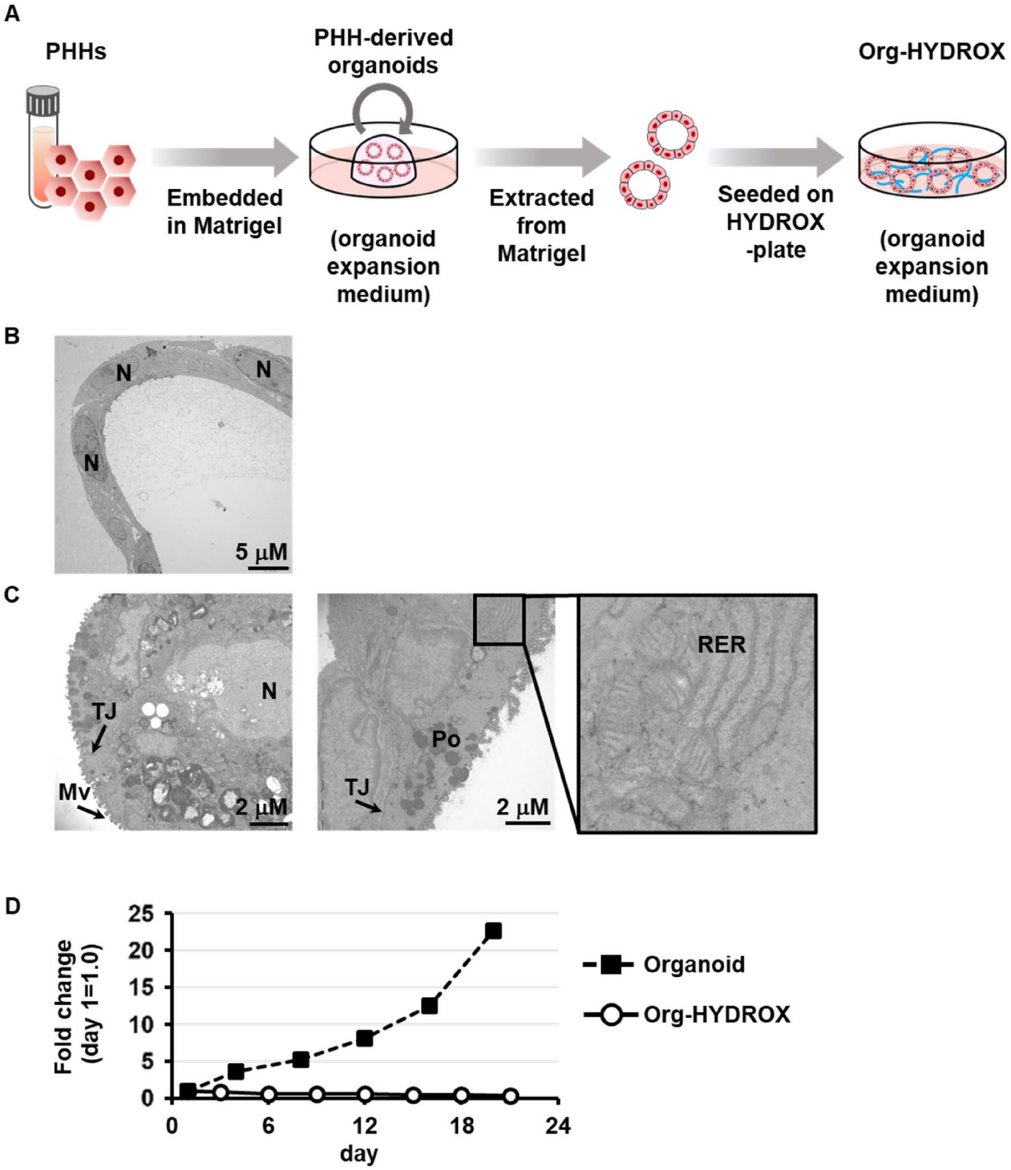
Histological characteristics of Org-HYDROX. (**A**) The procedure for culturing PHH-derived organoids using the HYDROX-culture system is presented schematically. Details of the cultivation method are described in the Materials and Methods. (**B, C**) Transmission electron microscope images of PHH-derived organoids (Organoid) (**B**) and Org-HYDROX (**C**) at day 10 of each culture condition (N: nuclear, TJ: tight junction, Mv: microvilli, Po: peroxisome, RER: rough endoplasmic reticulum). (**D**) The growth of PHH-derived organoids (Organoid) or Org-HYDROX was evaluated by CellTiter-Glo® 3D Cell Viability Assay. Data are presented as fold change (means ± S.D.) relative to day 0.

To perform HYDROX-culture, PHH-derived organoids were extracted from Matrigel and seeded on HYDROX-coated plates (HYDROX-plates) as described in Fig. 1A. During the HYDROX-culture, PHH-derived organoids aggregated and reconstructed 3D spherical structures (Fig. S2). We examined the cellular structure of PHH-derived organoids and Org-HYDROX by transmission electron microscopy and found that various intracellular organelles including rough endoplasmic reticula, peroxisomes, and tight junctions were observed in Org-HYDROX (Figs. 1C, S3B,C), while none were observed in PHH-derived organoids (Figs. 1B, S3A). These results indicated that Org-HYDROX had different properties from PHH-derived organoids.

We then clarified that the proliferative ability of Org-HYDROX was low, while PHH-derived organoids had high proliferative potential (Fig. 1C). To further characterize the proliferative ability of Org-HYDROX, we examined the gene expression level of a Wnt-target gene (l*eucine-rich repeat-containing G-protein-coupled receptor 5 (LGR5)*) generally used as a marker for adult stem cells. The results showed that the gene expression level of *LGR5* in Org-HYDROX was significantly lower than that in PHH-derived organoids (Fig. S4). In addition, Org-HYDROX showed a lower expression level of *Ki67,* a cell proliferation marker, compared to PHH-derived organoids (Fig. S4). Collectively, these results suggested that PHH-derived organoids could be successfully cultured using HYDROX but lost their proliferative potential.

### Hepatic functions of Org-HYDROX

**Figure 2:**
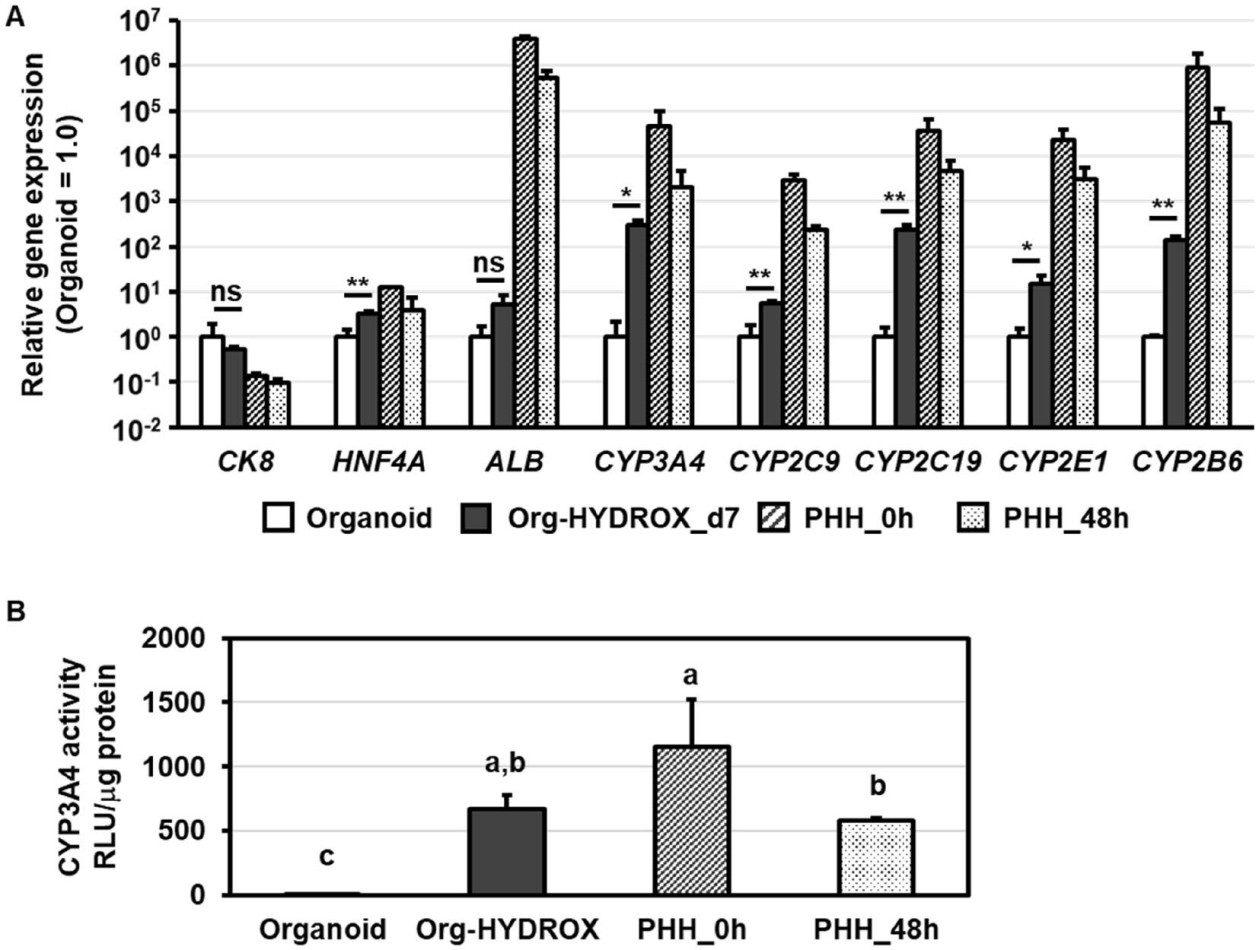
Hepatic functions of Org-HYDROX. (**A**) The gene expression levels of hepatocyte markers (*CK8, HNF4a, ALB, CYP3A4, CYP2C9, CYP2C19, CYP2E1, CYP2B6*) in PHH-derived organoids (Organoid) or Org-HYDROX cultured on HYDROX plates for 7 days. Parental PHHs, which were used for the generation of PHH-derived organoids and cultured for 0 h (just after thawing) or 48 h, were used as a positive control. The gene expression level of PHH-derived organoids (Organoid) was taken as 1.0. All data are represented as the means ± SD (*n* = 3). *p < 0.05, **p < 0.01. (**B**) The CYP3A4 activities in PHH-derived organoids (Organoid), Org-HYDROX and PHHs were examined by a luminescent assay system. Parental PHHs, which were used for the generation of PHH-derived organoids and cultured for 0 h (just after thawing) or 48 h, were used as a positive control. Statistical significance was evaluated by one-way ANOVA followed by Tukey’s post-hoc tests to compare all groups. Groups that do not share the same letter are significantly different from each other (*p* < 0.05). Results are shown as the mean ± SD (n = 3). All data are represented as the means ± SD (n = 3).

As described above, PHH-derived organoids lost their proliferative potential by HYDROX-culture. Therefore, we hypothesized that the PHH-derived organoids matured toward hepatocytes by HYDROX-culture. The gene expression levels of hepatocyte markers (*hepatocyte nuclear factor 4 alpha (HNF4a), CYP3A4, CYP2C9, CYP2C19, CYP2E1, CYP2B6*) in the Org-HYDROX cultured for 7 days on HYDROX-plates were upregulated compared to those in PHH-derived organoids (Fig. 2A). However, the gene expression levels of *cytokeratin8 (CK8)* and *ALB* were not significantly increased by HYDROX-culture (Fig. 2A). Similar results were obtained when culturing PHH-derived organoids generated from different PHH line, DOO, using HYDROX (Fig. S5). Moreover, the CYP3A4 activity in Org-HYDROX increased significantly compared to that in PHH-derived organoids, comparable to those of PHHs cultured for 48 h (Fig. 2B). To confirm the qRT-PCR results above, we conducted a western blotting analysis of PHH-derived organoids or Org-HYDROX using CYP3A4, CYP2C19 and CYP2C9 antibody for hepatocyte markers. The results showed that the protein expression levels of CYP3A4, CYP2C19 and CYP2C9 were higher in Org-HYDROX than in PHH-derived organoids (Fig. S6). These results suggested that the hepatic functions of PHH-derived organoids tended to recover by HYDROX-culture, particularly with regard to drug metabolizing enzyme capacity.

### Comprehensive gene expression level analysis of Org-HYDROX

**Figure 3:**
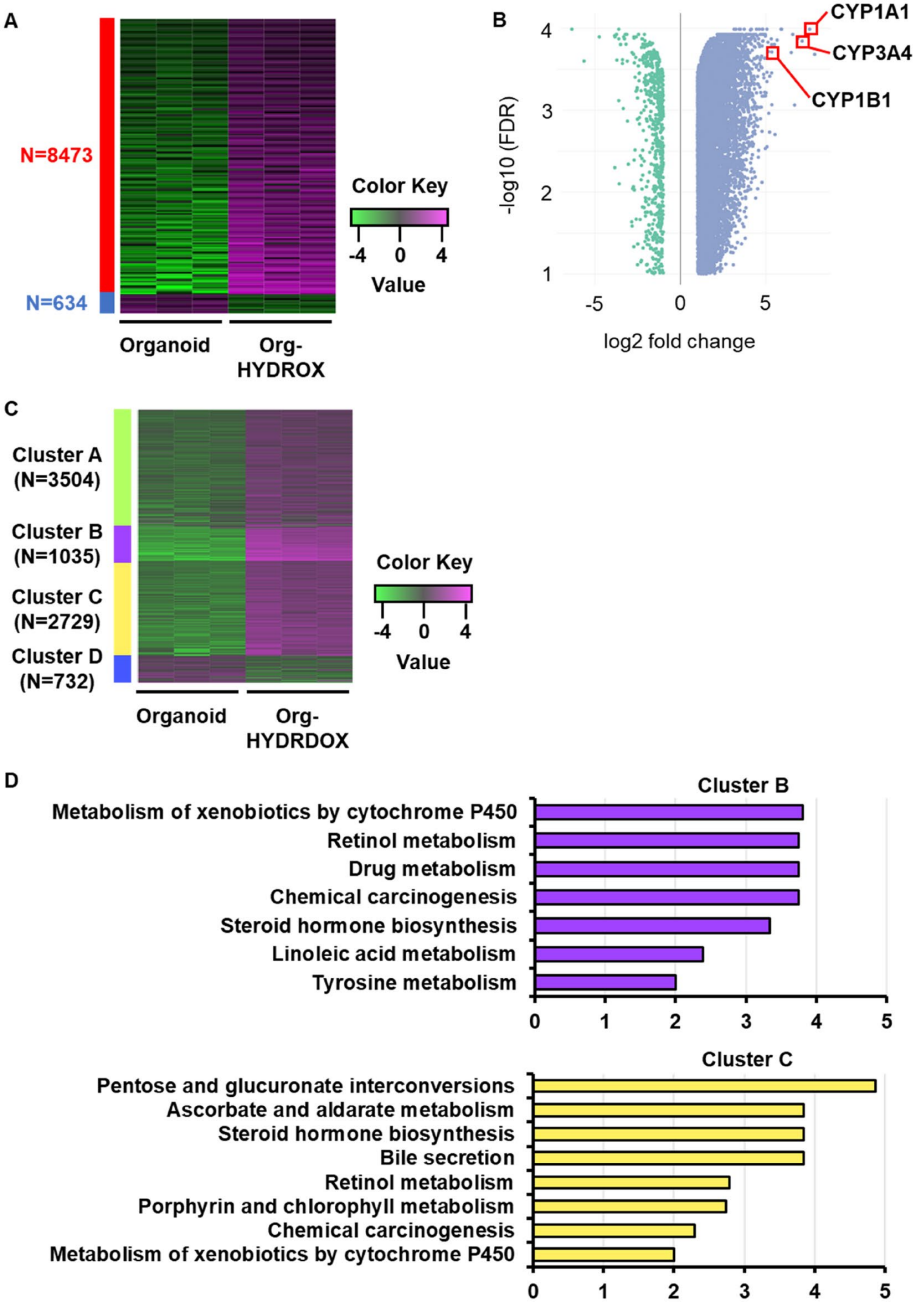
Comprehensive gene expression analysis of Org-HYDROX. (**A**) RNA-seq analysis was performed using PHH-derived organoids (Organoid) (n = 3) and Org-HYDROX (n = 3). The criteria for detection of differentially expressed genes (DEGs) in this study were FDR < 0.05 and a fold change > 2 (Org-HYDROX vs PHH-derived organoids (Organoid)). (**B**) Volcano plot showing genes differentially expressed in Org-HYDROX compared to PHH-derived organoids (Organoid). Blue dots represent upregulated genes, and green dots represent downregulated genes (FDR < 0.05, log_2_ |fold change|> 1). (**C**) K-means clustering was performed on DEGs, and the results are presented as a heatmap. (**D**) KEGG enrichment analysis was performed based on the functions of cluster B and cluster C obtained by k-means clustering.

Having determined that the hepatic function of PHH-derived organoids was increased by HYDROX-culture, we next performed a comprehensive gene expression analysis of PHH-derived organoids and Org-HYDROX. The 9000 most differentially expressed genes (DEGs) in Org-HYDROX versus PHH-derived organoids were analyzed. The results showed that 8473 genes were upregulated in Org-HYDROX against PHH-derived organoids (Fig. 3A). The top 20 DEGs upregulated in Org-HYDROX included drug metabolism-related genes such as CYP1A1, CYP3A4 and CYP1B1, suggesting that the hepatic functions of PHH-derived organoids were improved, especially with respect to drug metabolism (Fig. 3B), which is consistent with the results in Fig. 2. To analyze the gene expression in more detail, we performed k-means clustering, and 8000 genes were divided into 4 clusters (Fig. 3C). Clusters A, B and C showed highly expressed genes in Org-HYDROX compared to PHH-derived organoids, while cluster D showed the opposite pattern. Subsequently, each cluster was analyzed with regard to its biological function by KEGG enrichment analysis. As a result, processes related to hepatic functions, such as metabolism of xenobiotics by cytochrome P450, retinol metabolism, drug metabolism, steroid hormone biosynthesis and bile secretion, were found to be significantly enriched in clusters B and C (Fig. 3D). On the other hand, enrichment for processes related to the cell cycle was observed in cluster D, suggesting that PHH-derived organoids lost their proliferative potential as a result of the HYDROX-culture (Fig. S7). These results definitively confirmed that PHH-derived organoids lost their proliferative potential and improved their hepatic functions, especially those related to drug metabolism.

### Application of Org-HYDROX to drug-induced hepatotoxicity tests

**Figure 4:**
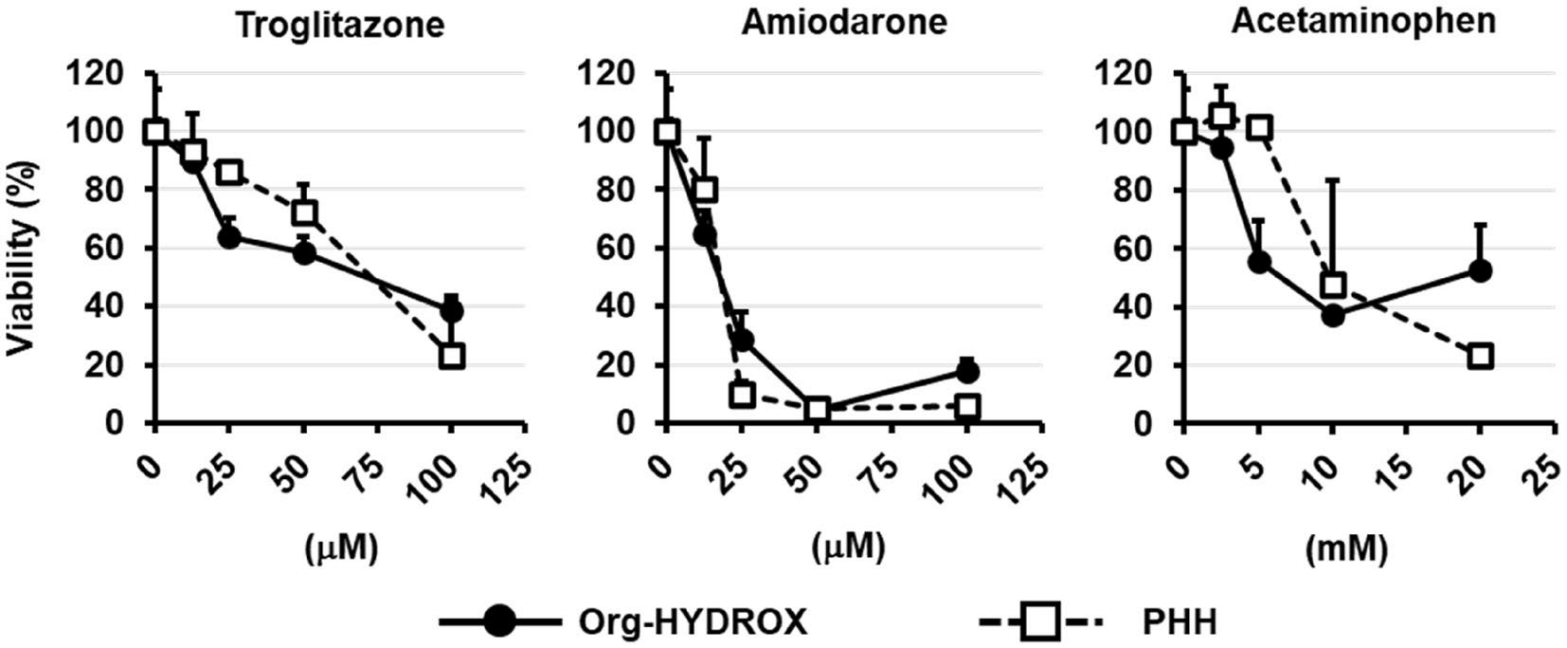
Application of Org-HYDROX to a drug-induced hepatotoxicity test. Org-HYDROX and PHHs (lot OHO; Celsis) after 48 h of culture were exposed to different concentrations of troglitazone, amiodarone and acetaminophen for 7 days. The cell viability of each cell type was examined by WST-8 assay and was calculated as a percentage of that in cells treated with vehicle only. All data are represented as the means ± SD (n = 3).

In the early stages of drug discovery, it is necessary to accurately estimate the safety of candidate compounds by means of a drug-induced hepatotoxicity test. Since Org-HYDROX exhibited high hepatic functions, including drug-metabolizing enzyme capacities, they are expected to be applicable as a novel hepatocyte model for pharmaceutical research. Thus, we examined the applicability of Org-HYDROX to a drug-induced hepatotoxicity test. The cell viability of Org-HYDROX when treated with different concentrations of three hepatotoxic drugs (troglitazone, amiodarone and acetaminophen) was measured. The cell viability of Org-HYDROX was found to be similar to that of PHHs (Fig. 4). These results suggested that Org-HYDROX could possibly be applied to drug-induced hepatotoxicity tests.

### Long-term culture of Org-HYDROX

**Figure 5:**
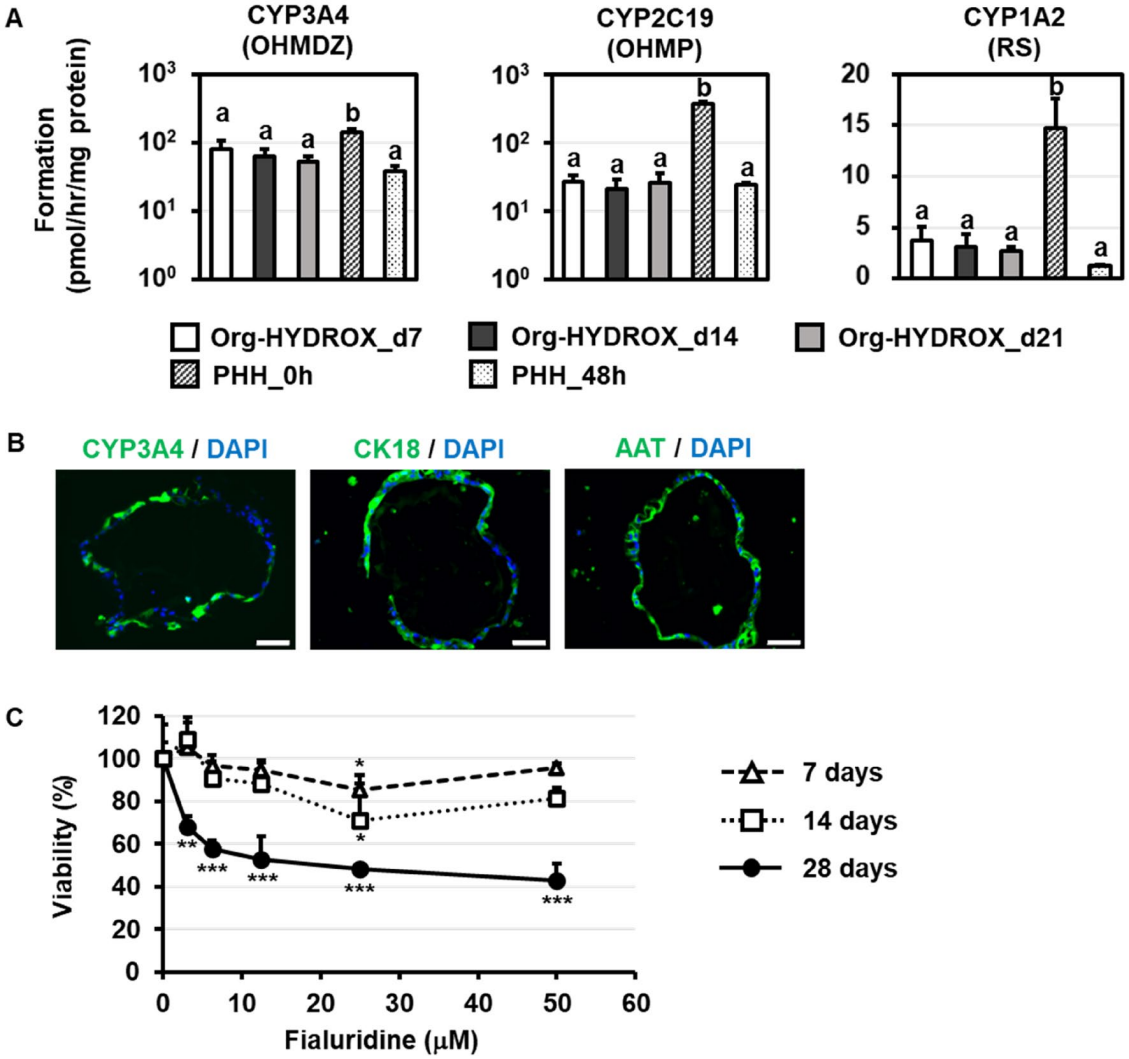
Long-term culture of Org-HYDROX. (**A**) The CYP3A4, CYP2C19 and CYP1A2 activities in PHH-derived organoids (Organoid), Org-HYDROX and PHHs were examined using LC–MS/MS by measuring the concentration of metabolites of the substrates for each CYP isozymes (OHMDZ, 1’-Hydroxy-midazolam; OHMP, (+/−)-4′-Hydroxy-mephenytoin; RS, Rerosufin). Parental PHHs, which were used for the generation of PHH-derived organoids and cultured for 0 h (just after thawing) or 48 h, were used as a positive control. Results are shown as the mean ± SD (n = 3). Statistical significance was evaluated by one-way ANOVA followed by Tukey’s post-hoc tests to compare all groups. Groups that do not share the same letter are significantly different from each other (*p* < 0.05). (**B**) The expressions of marker proteins for hepatocytes (CYP3A4, CK18, AAT) in Org-HYDROX were examined by immunostaining. Nuclei were counterstained with DAPI. Scale bars represent 50 µm. Images were acquired with fluorescence microscope (Biozero BZ-X800; KEYENCE). (**C**) PHH-derived organoids after 7 days of HYDROX-culture were treated with different concentrations of fialuridine. After 7, 14, and 28 days of fialuridine exposure, cell viability was examined by WST-8 assay and was calculated as a percentage of cells treated with vehicle only. All data are represented as the means ± SD (n = 3). *p < 0.05; **p < 0.01; ***p < 0.005.

In the drug development process, it is important to clarify the pharmacokinetics and toxicity of candidate compounds after long-term exposure from the viewpoint of safety assurance. In fact, attempts have been made to develop culture methods for long-term culture of PHHs^22–26^. Therefore, in this study, we investigated how the hepatic functions of Org-HYDROX fluctuate when cultured for a long period of time. We cultured PHH-derived organoids on HYDROX-plates up to 21 days, and then analyzed the gene expression levels of hepatocyte markers (*CK8, HNF4a, ALB, CYP3A4, CYP2C9, CYP2C19, CYP2E1, CYP2B6*) and the drug-metabolizing activities of CYP3A4, CYP2C19 and CYP1A2 at days 7, 14 and 21 of HYDROX culture (Fig. S8A, 5A). The gene expression levels and metabolic activities of each hepatocyte marker in Org-HYDROX increased along with the culture period, meaning that HYDROX could be used to culture highly functional PHH-derived organoids—i.e., with functionality partially comparable to PHHs—for at least 21 days. Furthermore, we performed immunocytochemistry and showed that Org-HYDROX expressed hepatocyte marker proteins including CYP3A4, CK18 and alpha-1 antitrypsin (AAT) at day 21 (Fig. 5B). In the case of *ALB*, however, gene expression did not reach that of PHHs, and protein expression was barely detected by immunocytochemistry (Fig. S8A,B). Furthermore, hepatic functions other than those related to drug metabolizing enzyme capacity were evaluated. We examined the gene expression levels of other hepatic markers such as xeno receptors (*pregnane X receptor (PXR), constitutive androstane receptor (CAR), farnesoid X receptor (FXR)*), hepatic transporters (Sodium + / taurocholate co-transporting polypeptide *(NTCP), breast cancer resistance protein (BCRP), multidrug resistance protein 2 (MRP2), multidrug resistance protein 1 (MDR1)*) and lipid metabolism (*sterol regulatory element-binding protein 1 (SREBP1), Peroxisome proliferator-activated receptor alpha (PPARA), apolipoprotein B (APOB), low-density lipoprotein receptor (LDLR)*) in PHH-derived organoids and Org-HYDROX (Figs. S9, S10, S11), and confirmed that these hepatic markers were significantly upregulated by HYDROX-culture. In addition, we compared the capacity of urea production in PHH-derived organoids and Org-HYDROX (Figure S12) and confirmed the increased urea production capacity in Org-HYDROX. These results altogether further demonstrated the efficient differentiation of PHH-derived organoids toward functional hepatocytes. Finally, in contrast to the maturation toward hepatocytes, we also confirmed the reduction in the cholangiocyte marker gene expressions (*EpCAM, CK7*) in Org-HYDROX (Fig. S13).

Given the finding that Org-HYDROX could maintain its high hepatic functions for a long period, except with respect to *ALB* expression, we next tested its ability to predict chronic drug-induced hepatotoxicity. PHH-derived organoids that have been cultured on HYDROX-plate for 7 days were treated with fialuridine, a drug known to exhibit chronic drug-induced hepatotoxicity^27,28^, and then the cell viability was measured. After 28 days of the fialuridine exposure, significant cell death was observed in Org-HYDROX in a fialuridine concentration-dependent manner. Importantly, the cell viability was higher at 7 and 14 days than at 28 days of fialuridine exposure (Fig. 5C), suggesting that longer exposure of the cells to fialuridine was required to reveal the cell toxicity. We also confirmed by qRT-PCR that PHH-derived organoids cultured for 35 days on HYDROX-plates still maintained their high liver function, as evidenced by their gene expression levels of drug metabolizing enzyme-related markers (Fig. S14). Based on these results, Org-HYDROX could be used to predict chronic drug-induced hepatotoxicity. Interestingly, there was a significant increase in the gene expression of *ALB* in Org-HYDROX cultured on HYDROX-plates for 35 days (Figure S14), which phenomenon was not observed when those cells were cultured on HYDROX-plates for 7, 14, or 21 days (Figs. 2A, 5A). Therefore, it is possible that restoration of the *ALB* gene expression would require a longer culture period compared with other hepatic markers. Finally, we examined the potential use of long-term cultured Org-HYDROX which was cultured on HYDROX-plates for 28 days for the drug-induced hepatotoxicity test (Fig. S15). The result indicated that long-term cultured Org-HYDROX was also able to be applied to drug-induced hepatotoxicity test.

## Discussion

In this study, we proposed a novel 3D culture and hepatic differentiation system for human liver organoids established from commercially available PHHs (PHH-derived organoids). The system uses HYDROX, 3D nanofibers based on an amphiphilic PSar-PLLA polymer. PHH-derived organoids seeded on HYDROX-plates formed cell aggregates and lost their proliferative potential (Fig. 1). On the other hand, they exhibited improved expression and metabolic activity levels of hepatocyte markers regarded to drug-metabolizing enzyme capacity (Figs. 2, 3, 5). We showed that Org-HYDROX could be applied to drug-induced hepatotoxicity tests with the same degree of accuracy as PHHs (Fig. 4). Furthermore, we demonstrated that Org-HYDROX could maintain its high hepatic functions for up to 35 days and could successfully predict chronic drug-induced hepatotoxicity caused by fialuridine (Fig. 5C). Consequently, we believe that HYDROX is a novel biomaterial that could be used to efficiently differentiate PHH-derived organoids towards hepatocyte-like cells.

Matrigel is the most commonly used matrix for organoid culture. However, it includes a heterogenous mixture extracted from Engelbreth-Holm-Swarm murine sarcomas, which has safety concerns such as immunogenic responses in clinical applications^29^. Matrigel also has high batch-to-batch variability (up to 50%)^10,12^, which may influence the reproducibility of in vitro experiments using organoids^30^. Synthetic matrices, on the other hand, are chemically-defined and their water content, rigidity, stiffness and other properties can be controlled as as required^31^, resulting in fewer immunogenic responses and high reproducibility in comparison to animal-derived Matrigel. Therefore, we believe that HYDROX, a synthetic and xeno-free biomaterial suitable for organoid culture and differentiation, would be beneficial for applying PHH-derived organoids to hepatotoxicity tests and other clinical research.

Org-HYDROX achieved hepatic function level similar to those in PHHs. However, the gene expression level of *ALB*, one of the most important hepatocyte functions, in organoids after 7, 14 or 21 days of HYDROX-culture was not at all comparable to that of PHHs (Figs. 2A, S8). Although higher gene expression of *ALB* was observed in Org-HYDROX cultured for 35 days (Fig. S14), it is likely that HYDROX is more beneficial for improving drug metabolizing enzyme capacity and less helpful for promoting ALB secretion capacity. In the future, we would like to work on imparting ALB secretion ability to Org-HYDROX efficiently.

The culture medium we used for culturing Org-HYDROX was reported as an organoid expansion medium, not a maturation or differentiation medium^4^. It has previously been reported that human liver organoids can be differentiated toward hepatocytes with ALB secretion capacity when cultured in differentiation medium containing Notch inhibitor N-[(3,5-difluorophenyl)acetyl]-l-alanyl-2-phenyl]glycine-1,1-dimethylethyl ester (DAPT)^5^, fibroblast growth factor 19 (FGF19)^32^, dexamethasone^33^ and bone morphogenetic protein 7 (BMP-7)^34^. In addition, removal of the growth stimuli R-spondin and forskolin has been reported to upregulate the gene expression of *ALB* in human liver organoids^4^. We believe that applying differentiation medium containing such components instead of the expansion medium may result in further improvement to the liver functions of Org-HYDROX, especially ALB secretion capacity.

Fialuridine was developed as a treatment for chronic hepatitis B virus infection. However, although fialuridine passed various pre-clinical safety assessments, the clinical trial failed due to the identification of human-specific chronic hepatotoxicity^27,28^. The hepatotoxicity was not predicted at the preclinical stage because conventional preclinical in vitro models such as PHHs were designed primarily to assess the risk of acute drug toxicity, as their hepatocyte functions decrease immediately with culture^35,36^. Therefore, the development of various in vitro models that are able to maintain the high hepatic functions over the long term and that could predict chronic drug-induced hepatotoxicity in pre-clinical trials has been rigorously pursued^37–39^. Sandwich culture of PHHs was effective in delaying the deterioration of hepatic functions, but did not prevent the decrease^39^. Spheroid culture of PHHs performed by using bioreactors or a hanging-drop system recapitulated the characteristics of liver, but was difficult to handle^37,38^. Our 3D culture system using HYDROX has fairly high usability, since only a simple process of adding the cell suspension to the dried gel-coated plate is needed to obtain cell aggregates. Org-HYDROX can be separated from the HYDROX with only centrifugation, allowing the subsequent assays to be performed easily and conveniently^21^. Therefore, due to its high usability and high hepatic functions that could be maintained up to 35 days, Org-HYDROX could be a promising in vitro model in the field of pharmaceutical research. However, the time dependent toxicity at high concentration of Fialuridine in our study appears shallow compared to previously published data^22,40^. Since the toxicity of fialuridine is reported to attribute to mitochondrial dysfunction^40^, the actual applicability of HYDROX for long-term toxicity studies must be comprehensively determined based on the results of other assessment such as LDH leakage and mitochondrial function.

To further improve the versability of Org-HYDROX, the inclusion of proliferative capacity is highly desired. Optimization of the culture medium of Org-HYDROX is a possible way to achieve this. Shang et al*.* conducted a high throughput screening and identified small molecules which induced functional proliferation of mature PHHs^41^. Zhang et al*.* developed a defined medium to promote the proliferation of PHHs for more than one month with 10,000-fold expansion, which expressed markers of both mature hepatocytes and liver progenitors^42^. Moreover, improvement of oxygen supply could be expected to enhance the proliferative capacity of human liver organoids. Schneeberger et al*.* proposed a spinner flask method which could efficiently expand human liver organoids derived from LGR5-positive adult stem cells, due to improved oxygenation in the spinner flasks^6^. Collectively, these findings may be beneficial for improving the proliferative potential of Org-HYDROX in the future. Hepatocyte gene expression is reported to vary across the liver lobule, with distinct expression profiles for periportal (zone 1), midzonal (zone 2), and pericentral (zone 3) hepatocytes^43^. Hepatocyte markers regarded to drug-metabolizing enzyme capacity, which we found to be most differentially expressed in Org-HYDROX, belong to zone 3 which exist in a low oxygen environment. In contrast, zone 2 characterized by liver functions including beta oxidation, gluconeogenesis, urea and protein synthesis, and lipid metabolism is existed in an oxygen rich environment^44^. Thus, improvement of oxygen supply could also be beneficial for further improvement in liver functions of Org-HYDROX.

In conclusion, we showed that Org-HYDROX could be a useful hepatocyte model in pharmaceutical research due to its high hepatic function. This technology might also be useful for regenerative medicine in the near future.

## Methods

### PHH-derived organoids culture

Two lots of PHHs (lots HC4-24; purchased from XENOTECH, DOO; purchased from Celsis) were used to generate organoids (Table S1). PHHs were washed with cold Advanced DMEM/F12 (Thermo Fisher Scientific) and spun at 400 g for 5 min. The cell pellet was mixed with Matrigel (growth factor reduced, Corning) and 1 × 10^4^ cells were seeded per well in a 24-well plate. After the Matrigel had solidified, 500 μl of organoid expansion medium was added to each well. The organoid expansion medium was prepared as described in a previous report^4^ where it was used to produce cholangiocyte organoids. Briefly, advanced DMEM/F12 was supplemented with 1% antibiotic antimycotic solution and 1% GlutaMAX (GIBCO), 10 mM HEPES (Nacalai Tesque), 2% B27 supplement (GIBCO), 1.25 mM N-Acetylcysteine (Sigma), 10 mM Nicotinamide (Sigma), 10 nM recombinant gastrin (Merk), 50 ng/ml EGF (R&D), 10% R-Spondin1 conditioned medium (homemade), 100 ng/ml recombinant human FGF10 (peprotech), 25 ng/ml recombinant human HGF (R&D), 5 μM A83-01 (Wako), and 10 μM Forskolin (Wako). During cultivation, the medium was refreshed every 3 days. For the establishment of PHH-derived organoids, the medium was supplemented with 25 ng/ml recombinant Noggin (R&D), 7.5 ng/mL recombinant Wnt3a (R&D) and 10 μM Y27632 (Wako) for the first 3–4 days. Passage was performed in a 1:3 split ratio once every 10–14 days. PHH-derived organoids passaged 5–12 times were used in all experiments.

### Dried gel preparation

PSar-PLLA polymer was synthesized as described previously^20^, with 109 sarcosine units and 33 lactic acid units (lot; 013-049-039-1) or 120 sarcosine units and 31 lactic acid units (lot; 013-046-067-1) per molecule. The degree of polymerization was determined by proton nuclear magnetic resonance (1H-NMR), matrix-assisted laser desorption/ionization time-of-flight mass spectrometry (MALDI-TOF MS), and gel permeation chromatography (GPC). Then, dried gels were prepared using a modified protocol described previously^20^. Briefly, PSar-PLLA polymers (10 mg) were dissolved in 1 mL of 95% ethanol (Wako) and then heated using a block incubator (Atto) at 70 °C for 5 min. Next, 0.3 mL of the 10 mg/mL polymer solution was poured into a 48-well culture plate (Corning). Dried gels were obtained by drying at room temperature overnight to remove ethanol solvents. These steps were performed in a sterile environment. These coated plates were stored at room temperature before use.

### Three-dimensional culture of PHH-derived organoids in HYDROX

We named and trademarked the 3D nanofibers HYDROX™ (Shimadzu). These nanofibers were formed by the addition of liquid solutions to dried gels composed of PSar-PLLA polymer. In this study, HYDROX derived from 10 mg/mL dried gels is referred to as 10 mg/mL HYDROX. PHH-derived organoids were seeded on a 10 mg/mL HYDROX-coated 48-well plate in the culture media described above.

### Phase contrast images

HYDROX-cultured organoids (Org-HYDROX) were observed with an inverted phase-contrast microscope (CKX41; Olympus). Images were captured with a computer-assisted digital camera (DP21; Olympus).

### Transmission electron microscope (TEM) images

PHH-derived organoids or Org-HYDROX were fixed in phosphate-buffered 2% glutaraldehyde for TEM observation. Post-fixation, dehydration, embedding, ultrathin sectioning, staining, and observation were performed at the Core Instrumentation Facility, Research Institute for Microbial Diseases, Osaka University.

### Cell proliferation assessment

The CellTiter-Glo® 3D Cell Viability Assay (Promega) was used to assess organoid growth and viability according to the manufacturer's instruction. Luminescence was detected using a TriStar2 LB942 (Berthold Technologies).

### Real-time RT-PCR

Total RNA was isolated using ISOGENE (NIPPON GENE). Using 500 ng of the total RNA, cDNA was synthesized with a SuperScript VILO cDNA synthesis kit (Thermo Fisher Scientific). qRT-PCR was performed with Fast SYBR Green master mix (Thermo Fisher Scientific) using a StepOnePlus real-time PCR system (Applied Biosystems). The relative quantitation of target mRNA levels was performed by using the 2^-ΔΔCT^ method. The values were normalized by those of the housekeeping gene, *glyceraldehyde 3-phosphate dehydrogenase* (*GAPDH*). PCR primer sequences (described in Table S2) were obtained from PrimerBank (https://pga.mgh.harvard.edu/primerbank/).

### Luminescent assay for CYP3A4 activity

For the luminescent assay for CYP3A4 activity in the cells, P450-Glo™ CYP3A4 Assay Kits (Promega) were used. 3 μM Luciferin-IPA was used for CYP3A4 substrates, and the incubation time was 1 h. The fluorescence activity was measured with a luminometer (Lumat LB 9507, Berthold) according to the manufacturer’s instructions. The CYP3A4 activity was normalized with the protein content per well.

### RNA-sequencing and FASTQ file processing

Library preparation was performed using a TruSeq stranded mRNA sample prep kit (Illumina, San Diego, CA) according to the manufacturer’s instructions. An MGIEasy Universal Library Conversion Kit (App-A) was used to convert the libraries for use with DNBSEQ. Sequencing was performed on a DNBSEQ-G400RS platform (MGI, Shenzhen, China) in 2 × 100 bp paired-end mode. Data were analyzed using iDEP (integrated Differential Expression and Pathway analysis) (http://bioinformatics.sdstate.edu/idep96/). The Gene Expression Omnibus (GEO) accession number for the microarray analysis is GEO: GSE239375.

### Drug-induced hepatotoxicity test

PHH-derived organoids were seeded on a 10 mg/mL HYDROX-coated 96-well plate. After 14 days of the HYDROX culture, Org-HYDROX were cultured with organoid expansion medium containing different concentrations of acetaminophen (Wako), troglitazone (Wako) and amiodaron (FUJIFILM Wako Pure Chemical) for 7 days. As a control group, PHHs (lot OHO; Celsis) cultured with hepatocyte culture medium (HCM; Lonza) for 48 h after plating on a 96-well plate were exposed to different concentrations of acetaminophen (Wako), troglitazone (Wako) and amiodaron (FUJIFILM Wako Pure Chemical) for 7 days. Cell viability was evaluated using a Cell Counting Kit-8 (Dojindo Laboratories) based on a WST-8 assay according to the manufacturer's instructions. The absorbance at 450 nm was determined by a multiplate reader (Bio-Rad). Cell viability was calculated as a percentage of the control (DMSO-treated group) viability, which was taken as 100%.

### UPLC-MS/MS analysis for the measurement of cytochrome P450 (CYP) activities

Ultra-performance liquid chromatography tandem mass spectrometry (UPLC-MS/MS) analysis was performed to examine the CYP3A4, CYP2C19 and CYP1A2 activities in the cells. PHH-derived organoids, Org-HYDROX and PHHs were cultured with medium containing each substrate shown in Table S3. After the treatment with substrates, the supernatant was collected at 24 h, and then immediately mixed with 2 volumes of acetonitrile (Wako). Samples were filtered with AcroPrep Advance 96-well filter plates (Pall Corporation), and then the supernatant was analyzed by UPLC-MS/MS to measure the concentration of metabolites according to each standard curve. UPLC analysis was performed using an Acquity UPLC (Waters) and MS/MS was performed on a Xevo TQ-S (Waters, Milford MA). LC separations were carried out at 40 °C with an Acquity UPLC BEH C18 column (1.7 μm, 2.1 × 50 mm; Waters). The mobile phase was delivered at a flow rate of 1.0 mL/min using a gradient elution profile consisting of solvent A (0.1% formic acid/distilled water) and solvent B (0.1% formic acid/acetonitrile). Five μL of sample solution was injected into the column. The gradient conditions were as follows: 0 min–2% B, 1.0 min–95% B, 1.25 min–95% B, 1.26 min–2% B, and 1.75 min–2% B. The concentrations of each metabolite were calculated according to each standard followed by normalization to the protein content per well.

### Immunocytochemistry

To perform the immunocytochemistry, Org-HYDROX washed with PBS were embedded in iPGell (GenoStaff) according to the manufacturer’s instructions, and then fixed with 4% paraformaldehyde for 15 min. After blocking the cells with PBS containing 2% bovine serum albumin (Nacalai Tesque) and 2% Triton X-100 (Merck) for 30 min at room temperature, the cells were incubated with the blocking buffer containing a primary antibody (described in Table S4) for 3 h at room temperature, and finally with the blocking buffer containing a secondary antibody (described in Table S4) for 1 h at room temperature. Nuclei were counterstained with 4’,6-diamidino-2-phenylindole (Nacalai Tesque). Images were acquired with fluorescence microscope (Biozero BZ-X800; KEYENCE).

### Statistical analysis

Statistical analyses were performed using the unpaired two-tailed Student’s t-test or one-way analysis of ANOVA, followed by Tukey’s post hoc tests. All data are represented as means ± SD. Statistical analyses were performed using GraphPad Prism. Details are described in the figure legends.

### Supplementary Information

Supplementary Information.

## Data Availability

The datasets generated and/or analyzed during the current study are available in the National Center for Biotechnology Information (NCBI) repository. The Gene Expression Omnibus (GEO) accession number for the microarray analysis is GEO: GSE239375 (scheduled to be released on Jan 31, 2025).
