# Supplementary material for: Efficient hepatocyte differentiation of primary human hepatocyte-derived organoids using three dimensional nanofibers (HYDROX) and their possible application in hepatotoxicity research
Source: Sci Rep. 2024 May 13;14:10846. doi: 10.1038/s41598-024-61544-y (PMC11089038; doi:10.1038/s41598-024-61544-y)
Supplement: Supplementary file 1 — Supplementary Information. [file 41598_2024_61544_MOESM1_ESM.docx]

**Supplemental Method**

**Western blotting**

The cells were homogenized with RIPA Lysis and Extraction buffer (Thermo Fisher Scientific) containing a protease inhibitor mixture (Sigma Aldrich). After being frozen and thawed, the homogenates were centrifuged at 15,000 × g at 4 °C for 15 min, and the supernatants were collected. The lysates were subjected to SDS-PAGE on 12.5% polyacrylamide gel and then transferred onto polyvinylidene fluoride membranes (Millipore). After the reaction was blocked with 5% skim milk in Tris-buffered saline (TBS) containing 0.1% Tween 20 at room temperature for 1 h, the membranes were incubated with primary antibodies at 4°C overnight, followed by reaction with secondary antibodies at room temperature for 1 h. The band was visualized by Chemi-Lumi One Super (Nakalai Tesque), and the signals were read using an LAS-3000 imaging system (FUJIFILM). All antibodies used in this report are described in **Table S5**.

**Urea secretion**

The culture supernatants, which were incubated for 72 h after fresh medium was added, were collected to determine their amount of urea production. A urea assay kit was purchased from BioAssay Systems. The experiment was performed according to the manufacturer’s instructions. The amount of urea secretion was calculated according to each standard followed by normalization to the protein content per well.

**Supplemental Tables**

**Table S1　Information for primary (cryopreserved) human hepatocytes (PHHs) used in this study**

| lot | HC4-24 | DOO | OHO |
| --- | --- | --- | --- |
| species | human | human | human |
| sex | Female | male | male |
| age | 22 | 57 | 28 |
| race | Caucasian | Caucasian | Caucasian |
| Post-thaw viability by trypan blue exclusion | 75% | 83% | 86% |
| Cause of death | Cerebrovascular accident | Anoxia; 2^nd^ to CVA | Head Trauma; 2^nd^ to GSW |

**Table S2 The primers used for real-time RT-PCR**

|  | for real time RT-PCR |
| --- | --- |
| Gene Symbol | Primers (forward/reverse; 5' to 3') |
| *ALB* | TGCAACTCTTCGTGAAACCTATG/ACATCAACCTCTGGTCTCACC |
| *APOB* | ACACACTGGACGCTAAGAGGA/ACTTGTGCTACCATCCCATACT |
| *BCRP* | TGCAACATGTACTGGCGAAGA/TCTTCCACAAGCCCCAGG |
| *CAR* | TAATGCGCTGACTTGTGAGG/TCATGCCAGCATCTAAGCAC |
| *CK7* | AGACGGAGTTGACAGAGCTG/GGATGGCCCGGTTCATCTC |
| *CK8* | TGAGGTCAAGGCACAGTACG/TGATGTTCCGGTTCATCTCA |
| *CK19* | CTCCCGCGACTACAGCCACT/ TCAGCTCATCCAGCACCCTG |
| *CYP2B6* | GCACTCCTCACAGGACTCTTG / CCCAGGTGTACCGTGAAGAC |
| *CYP2C9* | GGACAGAGACGACAAGCACA/CATCTGTGTAGGGCATGTGG |
| *CYP2C19* | TCTCTGTCCCAGCTCCAAGT/CAACAACCCTCGGGACTTTA |
| *CYP2E1* | ACCCGAGACACCATTTTCAG/TCCAGCACACACTCGTTTTC |
| *CYP3A4* | AAGTCGCCTCGAAGATACACA/AAGGAGAGAACACTGCTCGTG |
| *EpCAM* | AATCGTCAATGCCAGTGTACTT/TCTCATCGCAGTCAGGATCATAA |
| *FXR* | CACAGCGTTTTTGGTAATGC/TTGTTTGTGGAGACAGAGCCT |
| *GAPDH* | GGTGGTCTCCTCTGACTTCAACA/GTGGTCGTTGAGGGCAATG |
| *HNF4α* | CGTCATCGTTGCCAACACAAT/GGGCCACTCACACATCTGTC |
| *Ki67* | AGAAGAAGTGGTGCTTCGGAA/AGTTTGCGTGGCCTGTACTAA |
| *LDLR* | TCTGCAACATGGCTAGAGACT/TCCAAGCATTCGTTGGTCCC |
| *LGR5* | CTCCCAGGTCTGGTGTGTTG/GTGAAGACGCTGAGGTTGGA |
| *MDR1* | GCCAAAGCCAAAATATCAGC/TTCCAATGTGTTCGGCATTA |
| *MRP2* | TGAGCAAGTTTGAAACGCACAT/AGCTCTTCTCCTGCCGTCTCT |
| *NTCP* | AGAAGGTGGAGCAGGTGGT/ATCTTGGTCTGTGGCTGCTC |
| *PPARA* | GGCTGCAAGGGCTTCTTTCG/ACATCCCGACAGAAAGGCAC |
| *PXR* | TCCGGAAAGATCTGTGCTCT/AGGGAGATCTGGTCCTCGAT |
| *SREBP1* | TGCATTTTCTGACACGCTTC/CCAAGCTGTACAGGCTCTCC |

**Table S3 Substrates for CYP isozymes**

| Name | Substrate | Final concentration | Metabolite |
| --- | --- | --- | --- |
| CYP1A2 | Ethoxyresorufin | 0.5 μM | Rerosufin |
| CYP2C19 | (S)-(+)-Mephenytoin | 80 μM | (+/-)-4’-Hydroxy-mephenytoin |
| CYP3A4 | Midazolam | 1 μM | 1’-Hydroxy-midazolam |

**Table S4 The antibodies used for Immunohistochemistry**

| Antigen | Type | Company | Catalog number | Dilution factor |
| --- | --- | --- | --- | --- |
| CYP3A4 | rabbit | abcam | ab124921 | 1:200 |
| CK18 | mouse | invitrogen | MA5-12104 | 1:200 |
| ALB | goat | Bethyl laboratories | A80-129A | 1:200 |
| AAT | rabbit | Dako | A0012 | 1:200 |
| Alexa Fluor 488  anti-rabbit IgG | donkey | Thermo Fisher Scientific | A21206 | 1:1000 |
| Alexa Fluor 488  anti-mouse IgG | donkey | Thermo Fisher Scientific | A21202 | 1:1000 |
| Alexa Fluor 488 anti-goat IgG | donkey | Thermo Fisher Scientific | A11055 | 1:1000 |

**Table S5 The antibodies used for Western blotting**

| Antigen | Type | Company | Catalog number | Dilution factor |
| --- | --- | --- | --- | --- |
| CYP2C9 | rabbit | abcam | ab150364 | 1:200 |
| CYP2C19 | rabbit | NOSAN corporation | 130505 | 1:200 |
| CYP3A4 | rabbit | abcam | ab124921 | 1:400 |
| β-actin | mouse | Invitrogen | MA5-12104 | 1:200 |
| Anti-rabbit IgG, Peroxidase Antibody | goat | Cell signaling technology | 7074S | 1:1000 |
| Anti-mouse IgG, Peroxidase Antibody | horse | Cell signaling technology | 7076S | 1:1000 |

**Supplemental Figures**

**
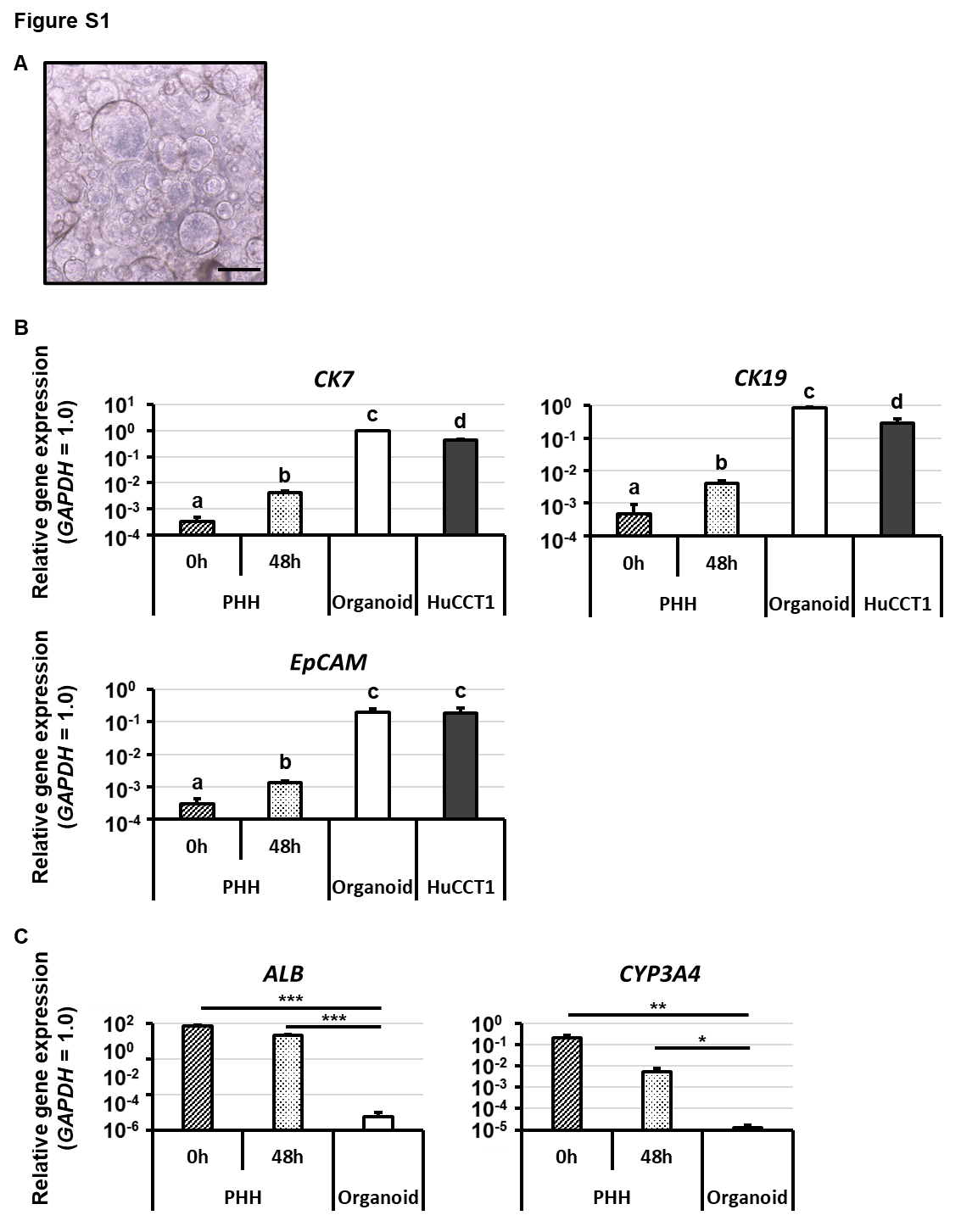
**

**Figure S1 Characterization of PHH-derived organoids**

**Organoids** established from PHHs (lot HC4-24) were cultured with organoid expansion medium. (**A**) Phase contrast microscopic image of PHH-derived organoids. Scale bars represent 500 µm. (**B**) The gene expression levels of cholangiocyte markers (*CK7, CK19, EpCAM*) in PHH-derived organoids (Organoid) and parental PHHs, which were used for the generation of PHH-derived organoids and cultured for 0 h (just after thawing) or 48 h. Human bile duct carcinoma cells (HuCCT1) were used as positive controls. The gene expression level of *GAPDH* was taken as 1.0. All data are represented as the means ± SD (n=3). Groups that do not share the same letter are significantly different from each other (p < 0.05). (**C**) The gene expression levels of hepatocyte markers (*ALB, CYP3A4*) in PHH-derived organoids (Organoid). Parental PHHs, which were used for the generation of PHH-derived organoids and cultured for 0 h (just after thawing) or 48 h, were used as a positive control. The gene expression level of *GAPDH* was taken as 1.0. All data are represented as the means ± SD (n=3). *p < 0.05, **p< 0.01, ***p<0.001.

**
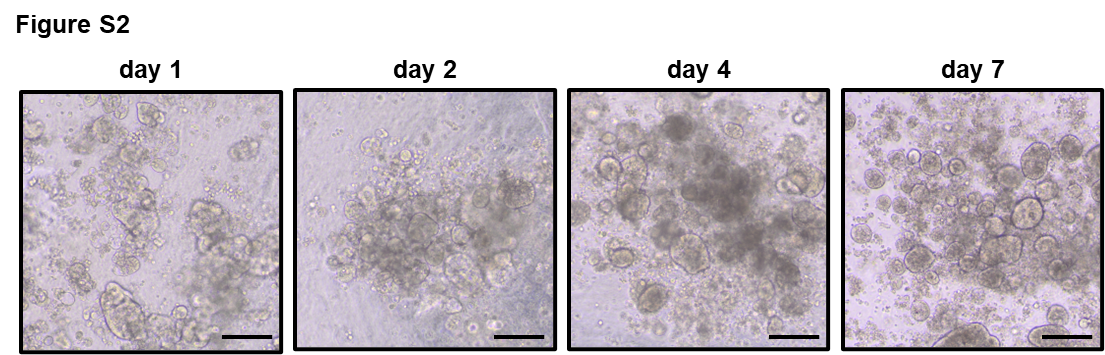
**

**Figure S2 Histological characteristics of Org-HYDROX**

PHH-derived organoids were extracted from Matrigel and seeded on HYDROX-coated plates. Phase contrast images of Org-HYDROX at day 1, 2, 4, and 7 of culture on HYDROX-plates are shown. Scale bars represent 500 µm.

**
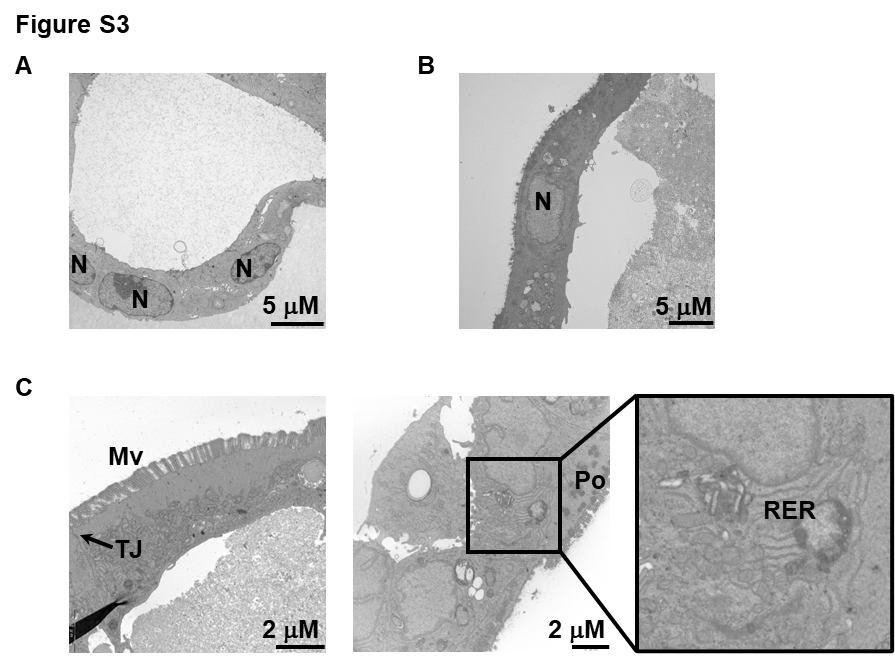
**

**Figure S3 Histological characteristics of Org-HYDROX**

Additional transmission electron microscope images of (A) PHH-derived organoids (Organoid) and (B,C) Org-HYDROX at day ten of each culture condition (N: nuclear, TJ: tight junction, Mv: microvilli, Po: peroxisome, RER: rough endoplasmic reticulum).

**
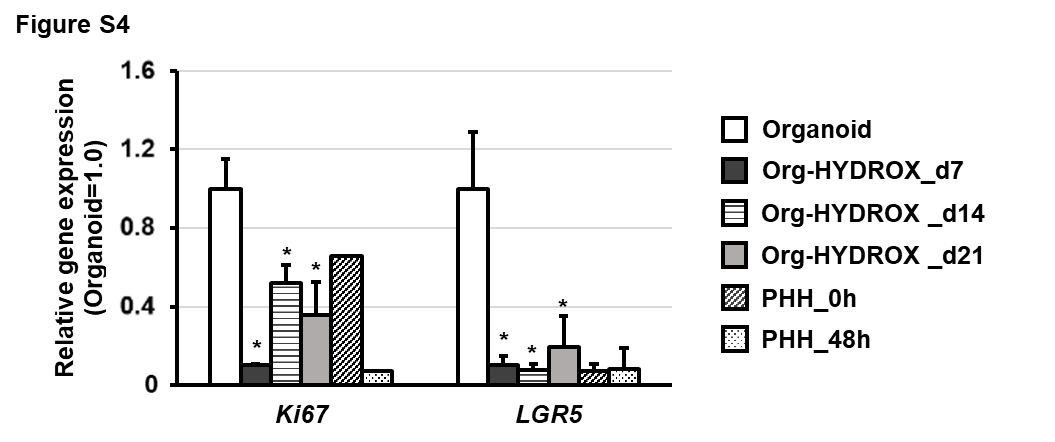
**

**Figure S4 Proliferative potential of Org-HYDROX**

The gene expression levels of a Wnt-target gene (*LGR5*) and a cell proliferation marker (*Ki67*) in PHH-derived organoids (Organoid) or Org-HYDROX were examined. Parental PHHs, which were used for the generation of PHH-derived organoids and cultured for 0 h (just after thawing) or 48 h, were used as a positive control. The gene expression level of PHH-derived organoids (Organoid) was taken as 1.0. All data are represented as the means ± SD (*n=*3). **p* < 0.05 (vs PHH-derived organoids).

**
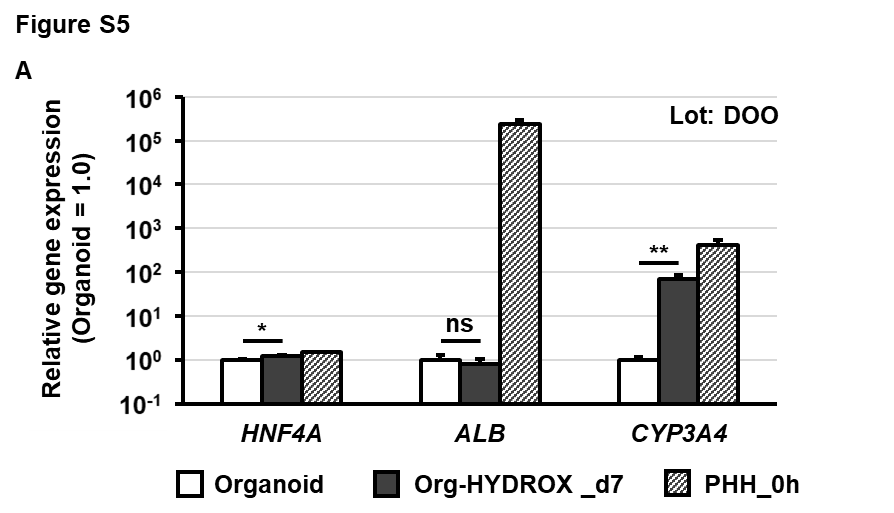
**

**Figure S5 PHH-derived organoids from a different PHH line**

Organoids established from PHHs (lot DOO) were cultured on HYDROX-plates as described in **Figure 1A**. The gene expression levels of hepatocyte markers (*HNF4a, ALB, CYP3A4*) were examined after 7 days of HYDROX-culture. Parental PHHs, which were used for the generation of PHH-derived organoids and cultured for 0 h (just after thawing), were used as a positive control. The gene expression levels in PHH-derived organoids (Organoid) were taken as 1.0. All data are represented as the means ± SD (*n=*3). **p* < 0.05, ***p* < 0.01, ns; not significant.

**
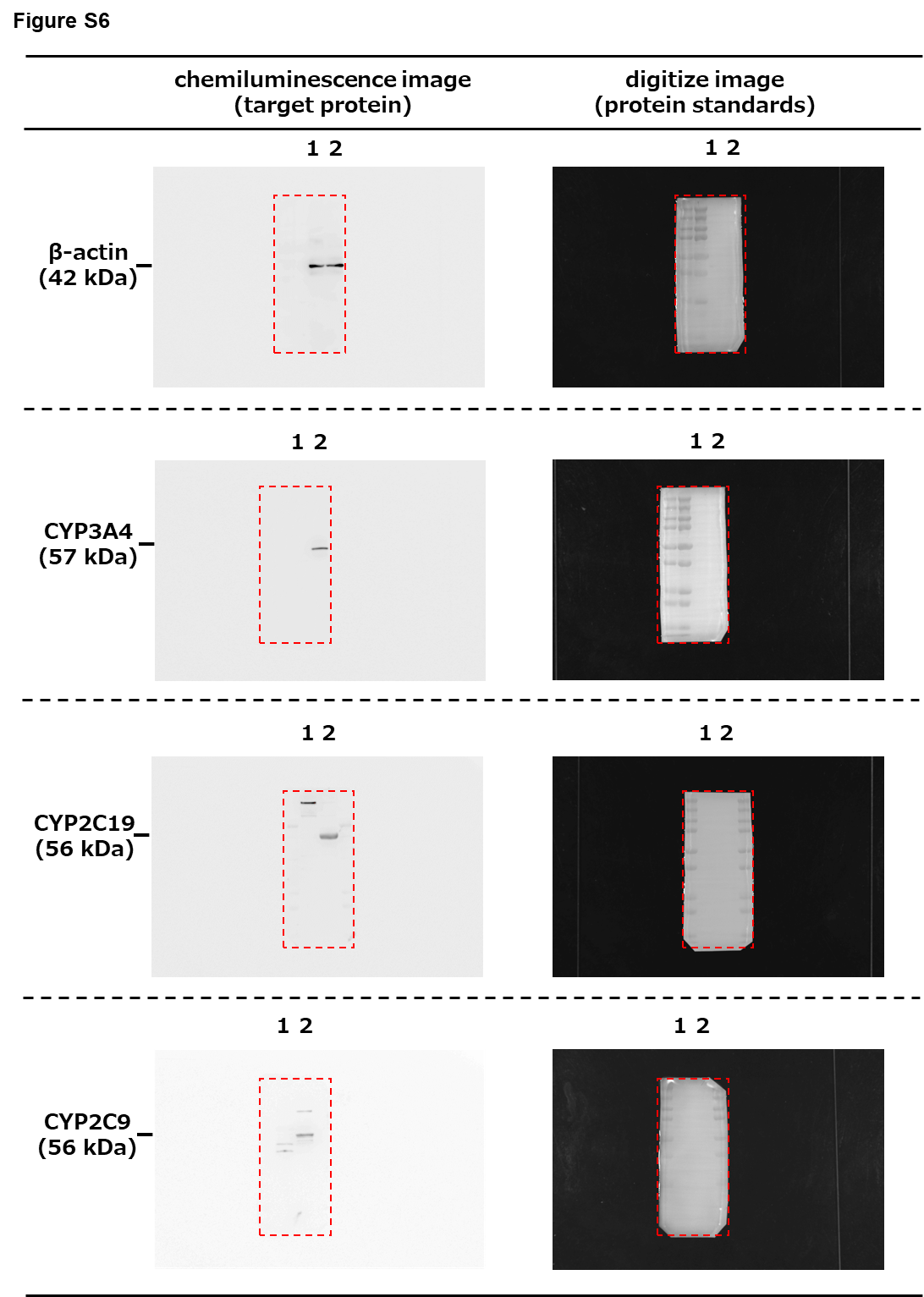
**

**Figure S6 Protein expression levels in Org-HYDROX**

The protein expression levels of hepatocyte markers, CYP3A4, CYP2C19, and CYP2C9 in PHH-derived organoids (lane 1) and Org-HYDROX cultured on HYDROX-plates for 7 days (lane 2) were examined by western blotting, with β-actin used as an internal control. For each target protein, chemiluminescence and digitized images of the same membrane in the full-length blot (red dotted lines) are shown. Chemiluminescence images indicates the expression of target protein, and digitize images shows the protein standards. All antibodies used are described in **Table S5**.

**
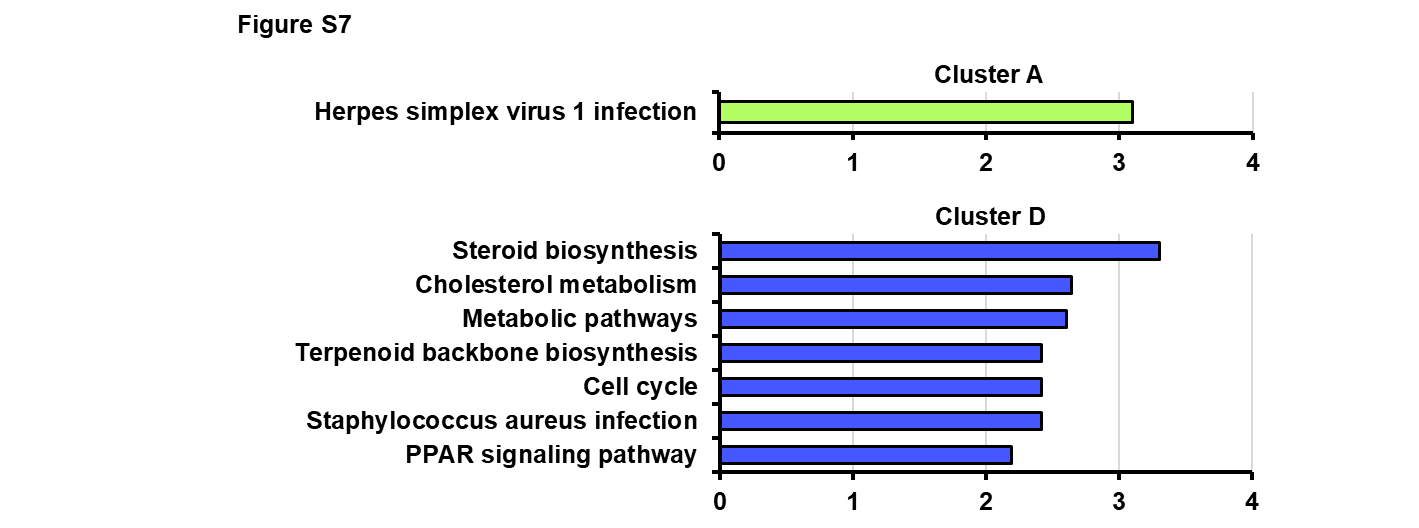
**

**Figure S7 Significantly enriched KEGG terms detected by KEGG enrichment analysis of each gene cluster**

RNA-seq analysis was performed using PHH-derived organoids (n=3 replicates) and Org-HYDROX (n=3 replicates). K-means clustering was performed on differentially expressed genes (FDR <0.05 and a fold change >2, Org-HYDROX vs PHH-derived organoids), and KEGG enrichment analysis was performed based on the functions of cluster A and cluster D.

**
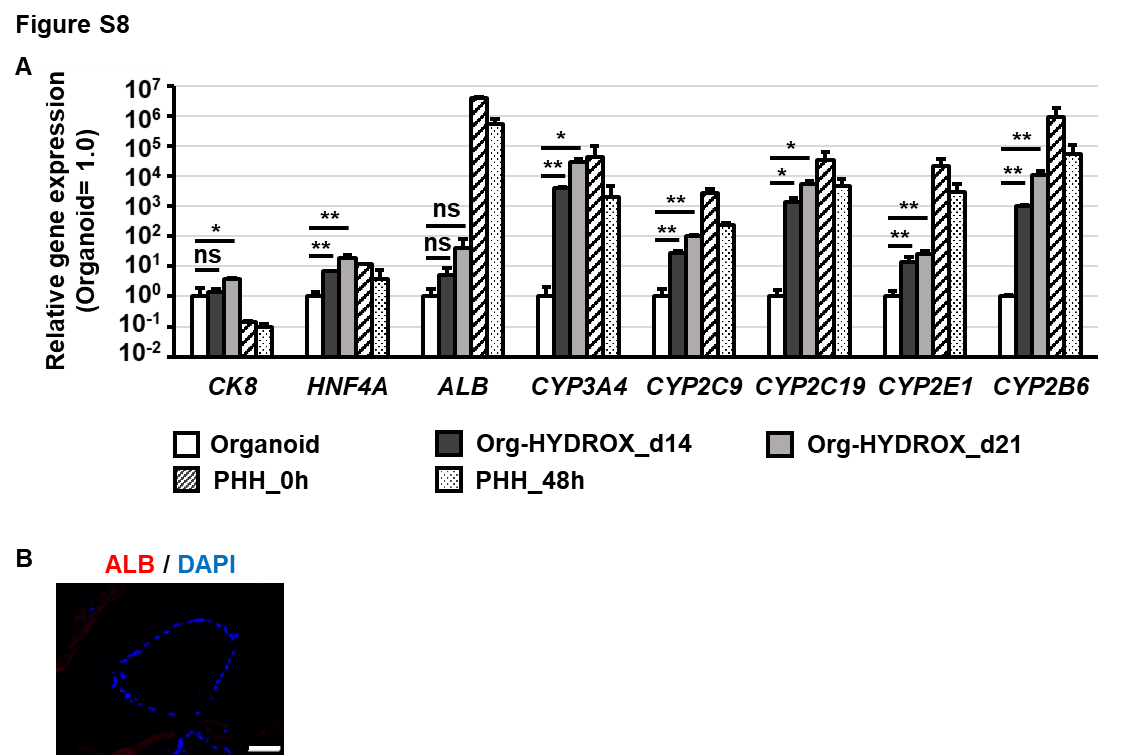
**

**Figure S8 Characterization of long-term cultured Org-HYDROX**

(**A**) The gene expression levels of hepatocyte markers (*CK8, HNF4a, ALB, CYP3A4, CYP2C9, CYP2C19, CYP2E1, CYP2B6*) in PHH-derived organoids (Organoid) and long-term (14 and 21 days) cultured Org-HYDROX were examined. Parental PHHs, which were used for the generation of PHH-derived organoids and cultured for 0 h (just after thawing) or 48 h, were used as a positive control. The gene expression level of PHH-derived organoids (Organoid) was taken as 1.0. All data are represented as the means ± SD (*n=*3). **p* < 0.05, ***p* < 0.01. (**B**) The expression of marker protein for ALB in Org-HYDROX were examined by immunostaining. Nuclei were counterstained with DAPI. Scale bars represent 50 µm. Images were acquired with fluorescence microscope (Biozero BZ-X800; KEYENCE).

**
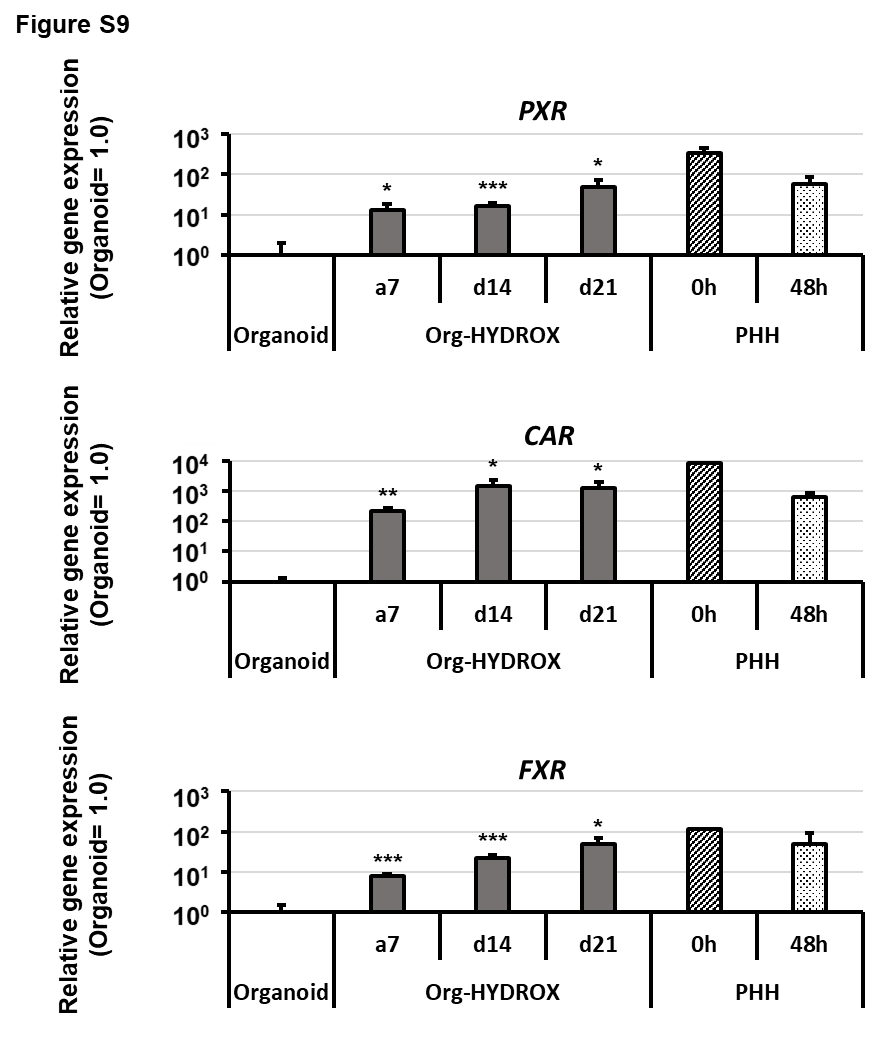
**

**Figure S9 Gene expression levels of Xeno receptor markers in Org-HYDROX cultured on HYDROX-plates for 7, 14, 21 days**

The gene expression levels of Xeno receptor markers (*PXR, CAR, FXR*) in PHH-derived organoids (Organoid) and Org-HYDROX cultured on HYDROX-plates for 7, 14 or 21 days were examined. Parental PHHs, which were used for the generation of PHH-derived organoids and cultured for 0 h (just after thawing) or 48 h, were used as a positive control. The gene expression level of PHH-derived organoids (Organoid) was taken as 1.0. All data are represented as the means ± SD (n=3). *p < 0.05, **p < 0.01, ***p<0.001.

**
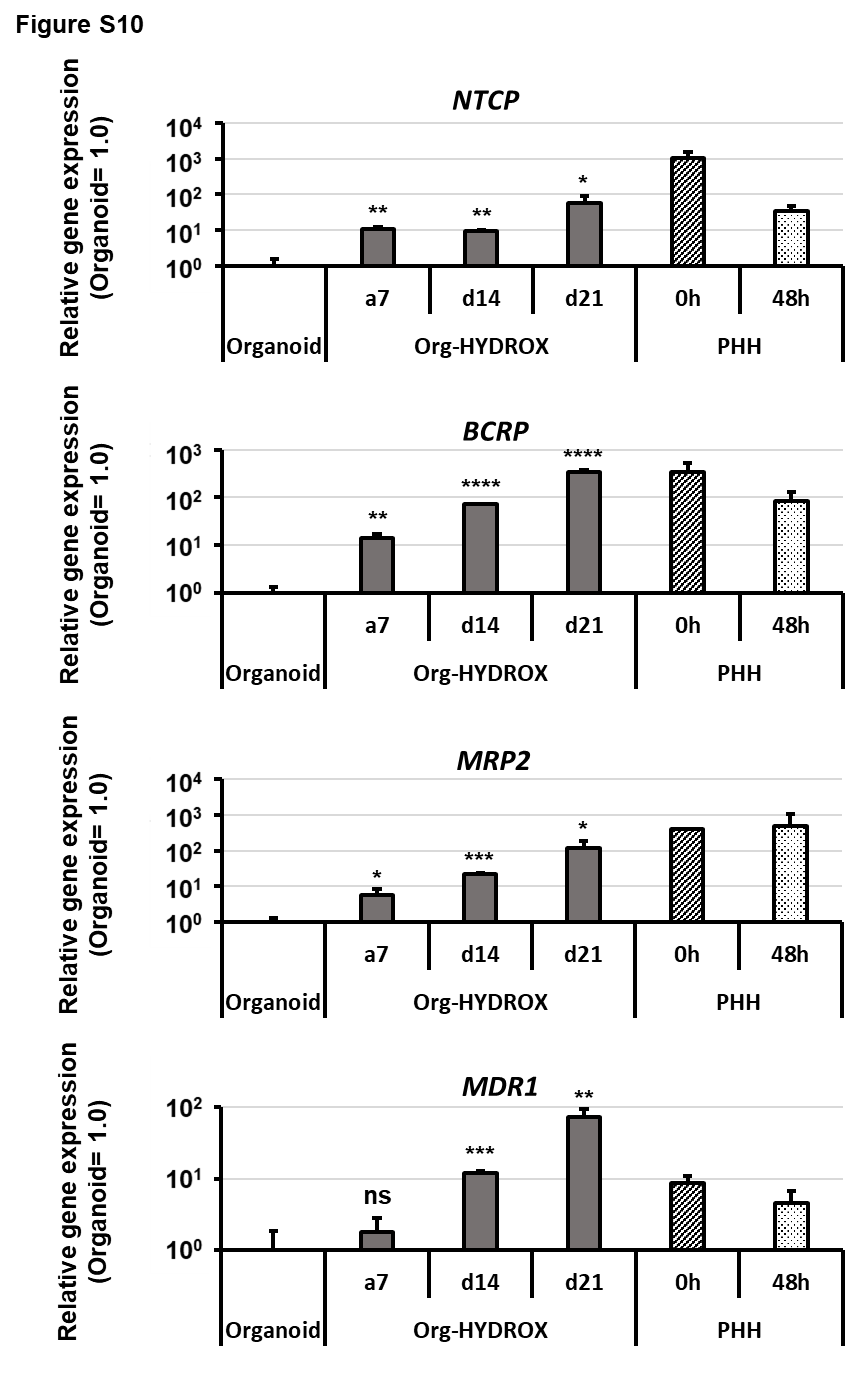
**

**Figure S10 Gene expression levels of hepatic transporter markers in Org-HYDROX cultured on HYDROX-plates for 7, 14, 21 days**

The gene expression levels of hepatic transporter markers (*NTCP, BCRP, MRP2, MDR1*) in PHH-derived organoids (Organoid) and Org-HYDROX cultured on HYDROX-plates for 7, 14 or 21 days were examined. Parental PHHs, which were used for the generation of PHH-derived organoids and cultured for 0 h (just after thawing) or 48 h, were used as a positive control. The gene expression level of PHH-derived organoids (Organoid) was taken as 1.0. All data are represented as the means ± SD (n=3). *p < 0.05, **p < 0.01, ***p<0.001, ****<0.0001, ns; not significant.

**
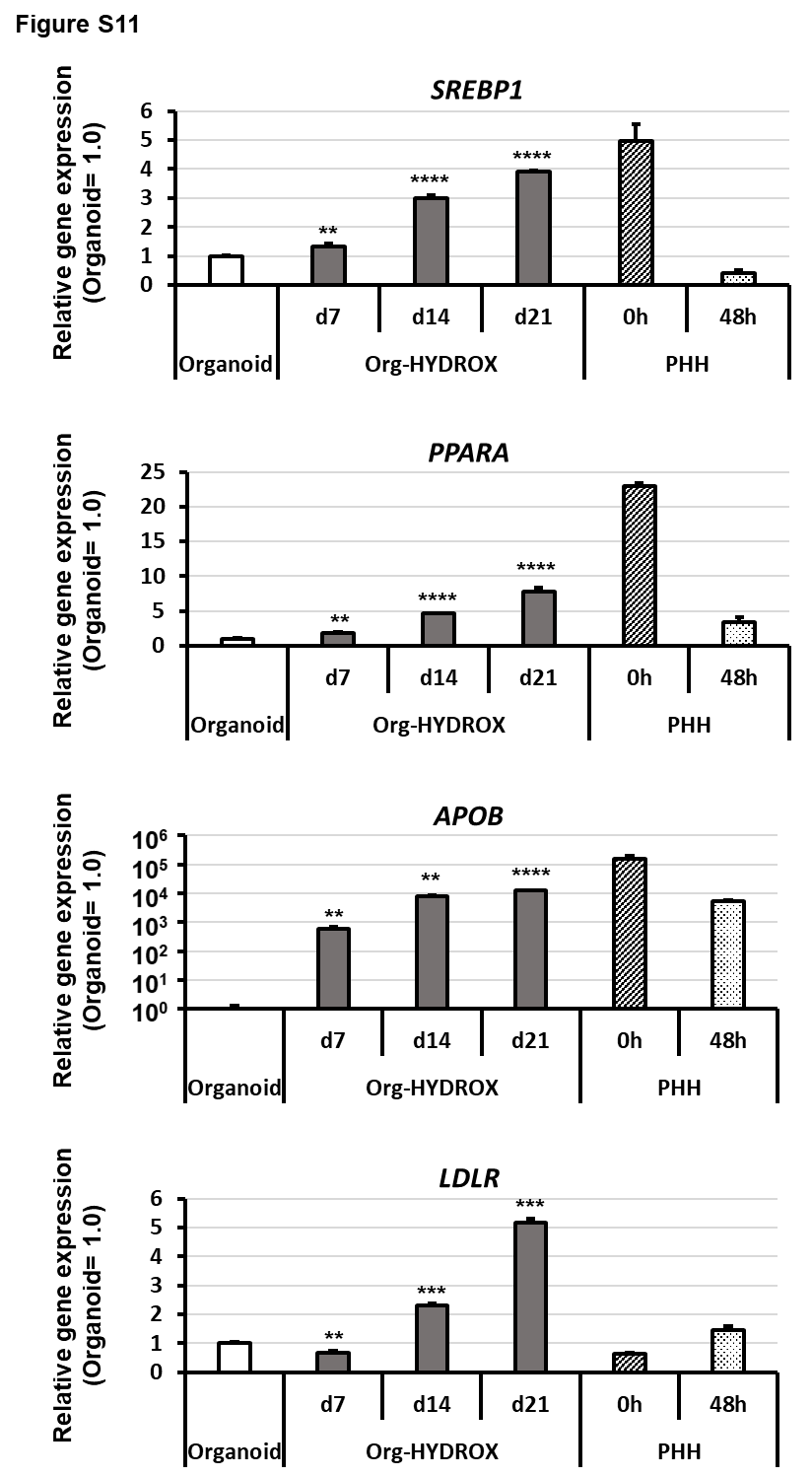
**

**Figure S11 Gene expression levels of lipid metabolism markers in Org-HYDROX cultured on HYDROX-plates for 7, 14, 21 days**

The gene expression levels of lipid metabolism markers (*SREBP1, PPARA, APOB, LDLR*) in PHH-derived organoids (Organoid) and Org-HYDROX cultured on HYDROX-plates for 7, 14 or 21 days were examined. Parental PHHs, which were used for the generation of PHH-derived organoids and cultured for 0 h (just after thawing) or 48 h, were used as a positive control. The gene expression level of PHH-derived organoids (Organoid) was taken as 1.0. All data are represented as the means ± SD (n=3). **p < 0.01, ***p<0.001, ****<0.0001.

**
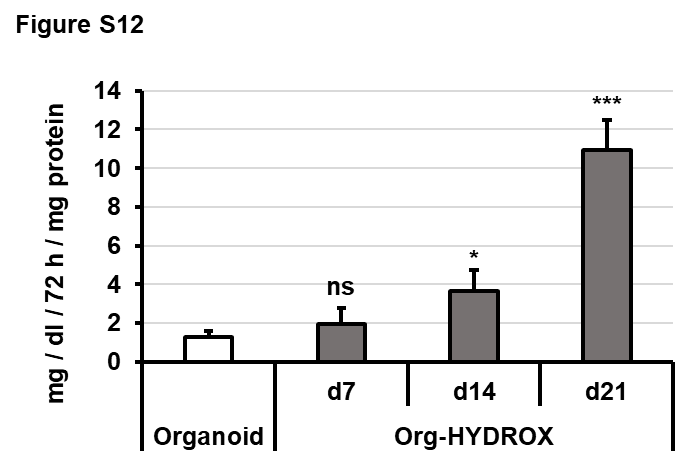
**

**Figure S12 Urea secretion ability of Org-HYDROX**

Production of urea in Org-HYDROX cultured on HYDROX plates for 7, 14 or 21 days were quantified. The amount of urea secretion was calculated according to each standard followed by normalization to the protein content per well. All data are represented as the means ± SD (n=3). *p<0.05, ***<0.001, ns; not significant.

**
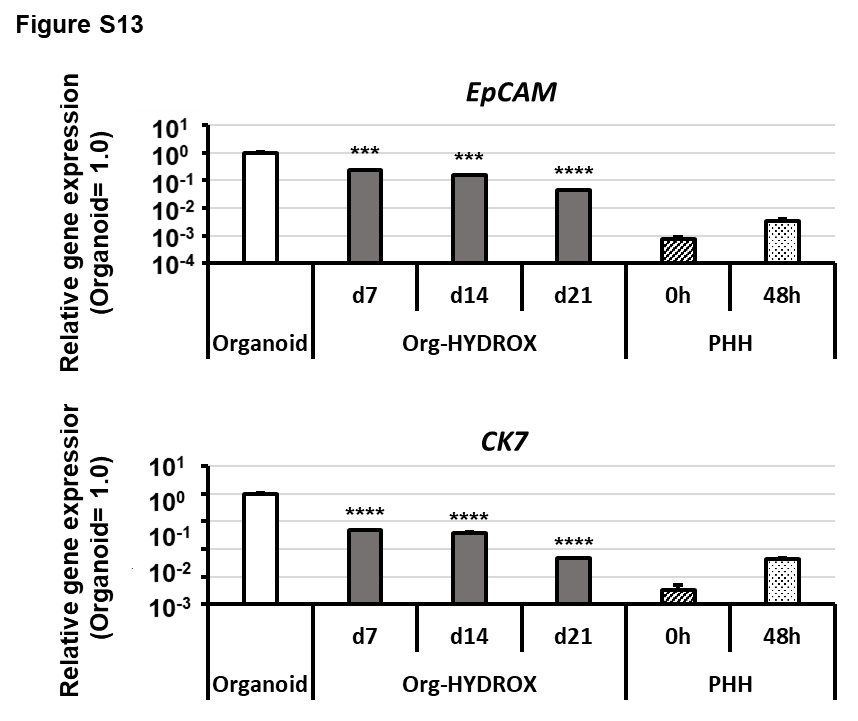
**

**Figure S13 Gene expression levels of cholangiocyte markers in Org-HYDROX cultured on HYDROX-plates for 7, 14, 21 days**

The gene expression levels of cholangiocyte markers (*EpCAM, CK7*) in PHH-derived organoids (Organoid) and Org-HYDROX cultured on HYDROX-plates for 7, 14 or 21 days were examined. Parental PHHs, which were used for the generation of PHH-derived organoids and cultured for 0 h (just after thawing) or 48 h, were used as a positive control. The gene expression level of PHH-derived organoids (Organoid) was taken as 1.0. All data are represented as the means ± SD (n=3). ***p<0.001, ****<0.0001.

**
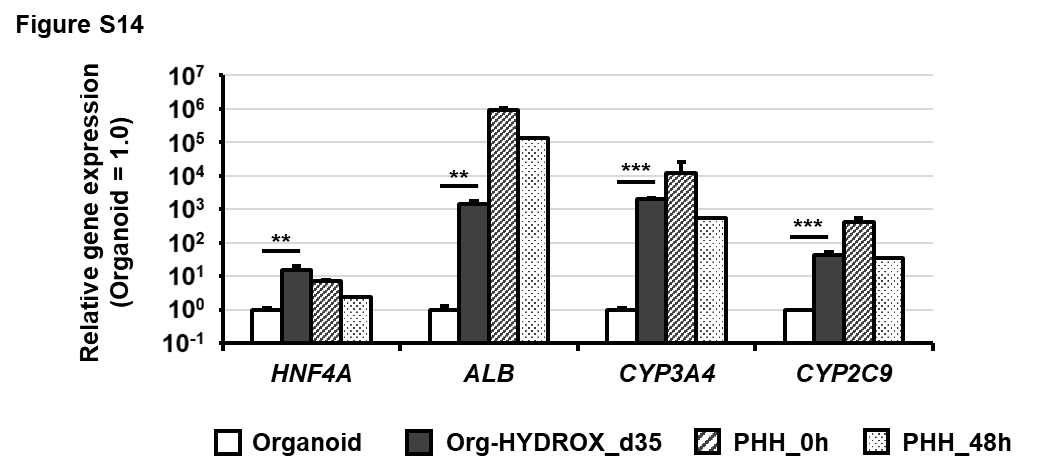
**

**Figure S14 Gene expression levels of Org-HYDROX cultured on HYDROX-plates for 35 days**

The gene expression levels of hepatocyte markers (*HNF4a, ALB, CYP3A4, CYP2C9*) in Org-HYDROX cultured on HYDROX-plates for 35 days were examined. Parental PHHs, which were used for the generation of PHH-derived organoids and cultured for 0 h (just after thawing) or 48 h, were used as a positive control. The gene expression levels in PHH-derived organoids (Organoid) were taken as 1.0. All data are represented as the means ± SD (*n=*3). ***p* < 0.01, ****p* < 0.005.

**
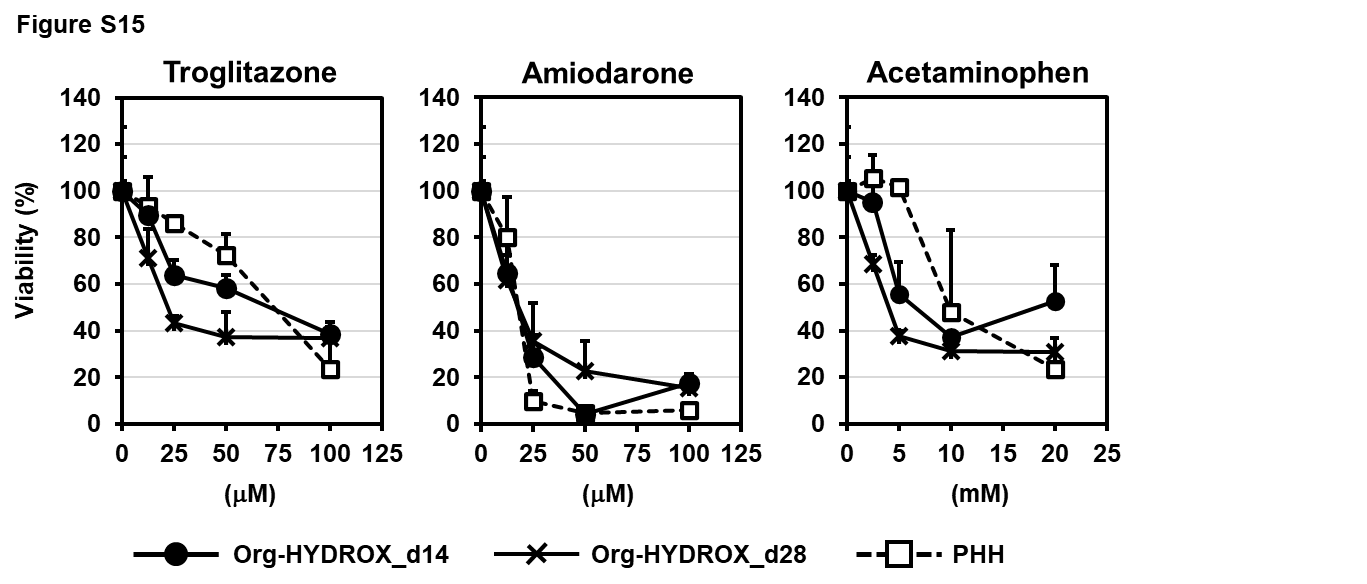
**

**Figure S15 Application of long-term cultured Org-HYDROX to a drug-induced hepatotoxicity test**

Org-HYDROX cultured on HYDROX plates for 14 or 28 days, and PHHs (lot OHO; Celsis) after 48 h of culture, were exposed to different concentrations of troglitazone, amiodarone and acetaminophen for 7 days. The cell viability of each cell type was examined by WST-8 assay and was calculated as a percentage of that in cells treated with vehicle only. All data are represented as the means ± SD (n=3).
